# Phenome-Wide Association Study (PheWAS) for Detection of Pleiotropy within the Population Architecture using Genomics and Epidemiology (PAGE) Network

**DOI:** 10.1371/journal.pgen.1003087

**Published:** 2013-01-31

**Authors:** Sarah A. Pendergrass, Kristin Brown-Gentry, Scott Dudek, Alex Frase, Eric S. Torstenson, Robert Goodloe, Jose Luis Ambite, Christy L. Avery, Steve Buyske, Petra Bůžková, Ewa Deelman, Megan D. Fesinmeyer, Christopher A. Haiman, Gerardo Heiss, Lucia A. Hindorff, Chu-Nan Hsu, Rebecca D. Jackson, Charles Kooperberg, Loic Le Marchand, Yi Lin, Tara C. Matise, Kristine R. Monroe, Larry Moreland, Sungshim L. Park, Alex Reiner, Robert Wallace, Lynn R. Wilkens, Dana C. Crawford, Marylyn D. Ritchie

**Affiliations:** 1 Center for Systems Genomics, Department of Biochemistry and Molecular Biology, The Pennsylvania State University, Eberly College of Science, The Huck Institutes of the Life Sciences, University Park, Pennsylvania, United States of America; 2 Center for Human Genetics Research, Vanderbilt University, Nashville, Tennessee, United States of America; 3 Information Sciences Institute, University of Southern California, Marina del Rey, California, United States of America; 4 Department of Epidemiology, University of North Carolina, Chapel Hill, North Carolina, United States of America; 5 Department of Genetics, Rutgers University, Piscataway, New Jersey, United States of America; 6 Department of Statistics, Rutgers University, Piscataway, New Jersey, United States of America; 7 Department of Biostatistics, University of Washington, Seattle, Washington, United States of America; 8 Division of Public Health, Fred Hutchinson Cancer Research Center, Seattle, Washington, United States of America; 9 Department of Preventive Medicine, Keck School of Medicine, University of Southern California/Norris Comprehensive Cancer Center, Los Angeles, California, United States of America; 10 National Human Genome Research Institute, National Institutes of Health, Bethesda, Maryland, United States of America; 11 Ohio State University, Columbus, Ohio, United States of America; 12 Epidemiology Program, University of Hawaii Cancer Center, Honolulu, Hawaii, United States of America; 13 University of Pittsburgh, Pittsburgh, Pennsylvania, United States of America; 14 Department of Epidemiology, University of Washington, Seattle, Washington, United States of America; 15 Departments of Epidemiology and Internal Medicine, University of Iowa, Iowa City, Iowa, United States of America; 16 Department of Molecular Physiology and Biophysics, Vanderbilt University, Nashville, Tennessee, United States of America; Georgia Institute of Technology, United States of America

## Abstract

Using a phenome-wide association study (PheWAS) approach, we comprehensively tested genetic variants for association with phenotypes available for 70,061 study participants in the Population Architecture using Genomics and Epidemiology (PAGE) network. Our aim was to better characterize the genetic architecture of complex traits and identify novel pleiotropic relationships. This PheWAS drew on five population-based studies representing four major racial/ethnic groups (European Americans (EA), African Americans (AA), Hispanics/Mexican-Americans, and Asian/Pacific Islanders) in PAGE, each site with measurements for multiple traits, associated laboratory measures, and intermediate biomarkers. A total of 83 single nucleotide polymorphisms (SNPs) identified by genome-wide association studies (GWAS) were genotyped across two or more PAGE study sites. Comprehensive tests of association, stratified by race/ethnicity, were performed, encompassing 4,706 phenotypes mapped to 105 phenotype-classes, and association results were compared across study sites. A total of 111 PheWAS results had significant associations for two or more PAGE study sites with consistent direction of effect with a significance threshold of p<0.01 for the same racial/ethnic group, SNP, and phenotype-class. Among results identified for SNPs previously associated with phenotypes such as lipid traits, type 2 diabetes, and body mass index, 52 replicated previously published genotype–phenotype associations, 26 represented phenotypes closely related to previously known genotype–phenotype associations, and 33 represented potentially novel genotype–phenotype associations with pleiotropic effects. The majority of the potentially novel results were for single PheWAS phenotype-classes, for example, for *CDKN2A/B* rs1333049 (previously associated with type 2 diabetes in EA) a PheWAS association was identified for hemoglobin levels in AA. Of note, however, *GALNT2* rs2144300 (previously associated with high-density lipoprotein cholesterol levels in EA) had multiple potentially novel PheWAS associations, with hypertension related phenotypes in AA and with serum calcium levels and coronary artery disease phenotypes in EA. PheWAS identifies associations for hypothesis generation and exploration of the genetic architecture of complex traits.

## Author Summary

In phenome-wide association studies (PheWAS) all potential genetic variants in a dataset are systematically tested for association with all available phenotypes and traits that have been measured in study participants. By investigating the relationship between genetic variation and a diversity of phenotypes, there is the potential for uncovering novel relationships between single nucleotide polymorphisms (SNPs), phenotypes, and networks of interrelated phenotypes. PheWAS also can expose pleiotropy, provide novel mechanistic insights, and foster hypothesis generation. This approach is complementary to genome-wide association studies (GWAS) that test the association between hundreds of thousands, to over a million, single nucleotide polymorphisms and a single phenotype or limited phenotypic domain. The Population Architecture using Genomics and Epidemiology (PAGE) network has measures for a wide array of phenotypes and traits, including prevalent and incident status for clinical conditions and risk factors, as well as clinical parameters and intermediate biomarkers. We performed tests of association between a series of genome-wide association study (GWAS)–identified SNPs and a comprehensive range of phenotypes from the PAGE network in a high-throughput manner. We replicated a number of previously reported associations, validating the PheWAS approach. We also identified novel genotype–phenotype associations possibly representing pleiotropic effects.

## Introduction

Phenomic approaches are complementary to the more prevalent paradigm of genome-wide association studies (GWAS), which have provided some information about the contribution of genetic variation to a wide range of diseases and phenotypes [1]. While a typical GWAS evaluates the association between the variation of hundreds of thousands, to over a million, genotyped single nucleotide polymorphisms (SNPs) and one or a few phenotypes, a common limitation of GWAS is the focus on a pre-defined and limited phenotypic domain. An alternate approach is that of PheWAS, which utilizes *all* available phenotypic information and all genetic variants in the estimation of associations between genotype and phenotype [1]. By investigating the association between SNPs and a diverse range of phenotypes, a broader picture of the relationship between genetic variation and networks of phenotypes is possible.

A challenge for PheWAS is the availability of large studies with genotypic data that are also linked to a wide array of high quality phenotypic measurements and traits for study. Biorepositories linked to electronic medical records (EMR) have been an initial resource for PheWAS, but these EMR-based studies are often limited to phenotypes and traits commonly collected for clinical use and may represent sets of limited racial/ethnic diversity [2], [3]. While there is no U.S. national, population-based cohort [4], several diverse, population-based studies exist with tens of thousands of samples linked to detailed survey, laboratory, and medical data. These large population-based studies have limitations [5], but collectively [6] they offer an opportunity to perform a PheWAS of unprecedented size and diversity.

To capitalize on the potential for collaborative discovery among some of the large population-based studies of the U.S., the National Human Genome Research Institute (NHGRI) funded the Population Architecture using Genomics and Epidemiology (PAGE) network. PAGE includes eight extensively characterized, large population-based epidemiologic studies where data were collected across multiple racial/ethnic groups, supported by a coordinating center [7], providing an exceptional opportunity to pursue PheWAS with a large number of SNPs, and thousands of phenotypic measurements including a wide range of common diseases, risk factors, intermediate biomarkers and quantitative traits in diverse populations. Herein, we illustrate the feasibility and utility of the PheWAS approach for large population-based studies and demonstrate that PheWAS provides information on, and exposes the complexity of, the relationship between genetic variation and interrelated and independent phenotypes. We have found PheWAS results that replicate previously identified genotype-phenotype associations with the exact phenotype in previous associations or closely related phenotypes, as well as a series of novel genotype-phenotype associations. This data exploration method exposes a more complete picture of the relationship between genetic variation and phenotypic outcome. PheWAS provides the unbiased, high throughput design achieved by GWAS in the genome and phenotype domains simultaneously. This approach changes the paradigm of phenotypic characterization and allows for exploratory research in both genomics and phenomics.

## Results

Data from five PAGE study sites were available for this PheWAS: Epidemiologic Architecture for Genes Linked to Environment (EAGLE) using data from the National Health and Nutrition Examination Surveys (NHANES); the Multiethnic Cohort Study (MEC); the Women's Health Initiative (WHI); and two studies of the Causal Variants Across the Life Course (CALiCo) group: the Cardiovascular Health Study (CHS) and Atherosclerosis Risk in Communities (ARIC). Text S1 provides full information on study design, phenotype measurement, and genotyping for each study. These studies collectively include four major racial/ethnic groups: European Americans (EA), African Americans (AA), Hispanics/Mexican Americans (H), and Asian/Pacific Islanders (API). All PAGE study sites included both males and females, except for WHI (which includes only women). Table 1 provides an overview of the sample sizes by PAGE study site as well as the number of SNPs and phenotypes available for this PheWAS. Sample size and the number of phenotypes varied across studies, and the sample size for various phenotypes within each study varied dependent on the number of individuals for which a given phenotype was measured. The number of phenotypes available for this PheWAS ranged within studies from 63 (MEC) to 3,363 (WHI). Study sites also had differing numbers of genotyped SNPs, and Table S1 contains the list of all SNPs available for two or more sites in this study, arranged by previously associated phenotypes. The PAGE network has focused on characterization of well-replicated variants across multiple race/ethnicities, so each study independently genotyped a set of SNPs with previously reported associations with phenotypes such as body mass index, C-reactive protein, and lipid levels.

**Table 1:** Study Descriptions.

				Maximum Sample Size^3^				Minimum Sample Size^3^						
Study^1^	PMID^2^	Age Range	Sex	EA	AA	H	API	EA	AA	H	API	# SNPs^4^	# Phenotypes^5^	Total # of Tests^6^ (# p<0.01)
ARIC	[36]	45–64	M/F	11,068	4,007	NA	NA	17	7	NA	NA	69	612	138,207 (2,378)
CHS	[37]	65–100 at baseline	M/F	4,487	820	NA	NA	151	116	NA	NA	46	341	34,829 (550)
EAGLE	[38]	12–95	M/F	2,628	2,107	2,071	NA	7	16	15	NA	236	327	359,508 (6,496)
MEC	[39]	45–75	M/F	3,893	4,749	6,863	6,810	33	27	40	13	74	63	23,310 (212)
WHI	[40]	50–79 at baseline	F	13,334	4,274	2,023	927	14	5	7	5	94	3,363	1,123,366 (14,068)

Abbreviations: European American (EA), African American (AA), Hispanic/Mexican American (H), Asian/Pacific Islander (API).

1 Data from PAGE studies available for this PheWAS include: Atherosclerosis Risk in Communities (ARIC), Cardiovascular Health Study (CHS), Epidemiologic Architecture for Genes Linked to Environment (EAGLE), Multiethnic Cohort (MEC), and Women's Health Initiative (WHI). PAGE study sites and study design descriptions are in Text S1.

2 Pubmed ID of study description manuscript for each study.

3 Maximum sample size and Minimum sample size are dependent both on who was genotyped and who had a specific phenotype measured. Not all phenotypic measurements were available for all participants within each study.

4 This is the total number of SNPs available for each study. Table S1 has the list of these SNPs for each study, genotyped across two or more studies.

5 This includes the number of phenotypes transformed and untransformed, as well as categorical phenotypes divided into binary phenotypes, full description in Materials and Methods.

6 Total number of tests of association calculated for each study, in parenthesis is the total number of associations with p<0.01.

Tests of association assuming an additive genetic model were performed independently by each PAGE study site for each SNP and each phenotype, stratified by race/ethnicity. The last column of Table 1 presents the total number of comprehensive associations with and without a p-value cutoff of 0.01, showing the proportion of significant results for this many tests of association. The total number of tests of association ranged from >20,000 (MEC) to >1 million (WHI) reflecting the variability in both the number of phenotypes available for study as well as the number of SNPs genotyped by each PAGE study site. As expected, the total number of significant tests of association (p<0.01) represented a fraction of the total number of tests performed.

Results from these tests of association were then compared across study sites to identify overlapping significant associations, as these results most likely represent robust findings. To facilitate determining overlapping significant associations, similar phenotypes that existed across more than one study were binned into 105 distinct phenotype-classes. For some phenotypes, the specific phenotype existed across more than one PAGE study, such as for the phenotype “Hemoglobin”, where hemoglobin measurements were available for ARIC, CHS, EAGLE, and WHI. Other groups of phenotypes binned within phenotype-classes were within similar phenotypic domains but were not represented in exact same form across studies. Table S2 contains a list of the study level phenotypes, the study from which the phenotype is available, and the phenotype-class for each phenotype that overlapped with another study.

The same or similar phenotypes may or may not have been collected by each PAGE study. Thus, the number of studies that were available for comparison of results across studies varied from one phenotype-class to another phenotype-class. Table 2 presents the number of results where at least two of five independent studies had SNP-phenotype associations with p<0.01 for single phenotype-class and single race/ethnicity group, compared to the total number of SNP-phenotype association tests performed. For example, >8,500 tests of association for the same SNP and same phenotype were available from two PAGE study sites whereas only 906 and 58 tests of association were available from four and five PAGE study sites, respectively. There were 3 results where two or more of the groups had a SNP–phenotype association p<0.01 for a single phenotype class across 5 groups represented.

**Table 2:** The number of SNP–Phenotype tests of association for phenotype-classes varies by PAGE study site genotype and phenotype overlap.

Number of PAGE study sites^1^	Number of Total Tests of Association^2^	Number of Significant Tests of Association^3^
2	8680	45
3	3295	42
4	906	21
5	58	3*
Total	12939	111

1 Number of PAGE study sites where both the SNP and phenotype were available for a given phenotype class.

2 Total number of tests of association, by number of PAGE study sites, where both the SNP and phenotype were available for a given phenotype class.

3 Number of tests of association that was significant (p<0.01) for two or more PAGE study sites for a single phenotype class and SNP, taking into account matching direction of effect when phenotypically relevant.

* Three results where two or more of the groups had a SNP–phenotype association p<0.01 for a single phenotype class across 5 groups represented. The most replicated novel results across studies were for SNPs rs599839, rs10923931, and rs2228145 with hematologic traits.

For this PAGE-wide PheWAS, tests of association were considered significant across PAGE study sites where two or more phenotypes in the same phenotype-class in the same racial/ethnic group passed a significance threshold of p<0.01 with a consistent direction of genetic effect. Based on these criteria, a total of 111 PheWAS associations were identified (Table S3). Overall, among the 111 significant PheWAS associations identified, 52 PheWAS results replicated previously published genotype-phenotype associations (Table S4), 26 represented phenotype-classes closely related to previously known genotype-phenotype associations (Table 3), and 33 represented novel genotype-phenotype associations (Table 4).

**Table 3:** PheWAS Tests of Association: Related Associations.

Nearest Gene	SNP ID	CA^1^, (CAF^2^)	Phenotypes^3^	Associated Phenotype Class^4^	Ethnicity^5^	P-Values^6^	Beta(SE)^7^	Sample Size^8^	Substudies^9^	Substudy Count^10^	Previously Associated Phenotype^11^	References^12^
CELSR2, PSRC1	RS599839	A (0.8)	Ever hospitalized for chest pain? (Y/N)	ANGINA	EA	2.57e-3	0.55(0.18)	568	ARIC	3	Coronary Artery Disease, LDL cholesterol, Total cholesterol	18262040, 18193043, 18179892, 17634449, 18193044
			Angina status at baseline (Y/N)			8.79e-3	0.21(0.08)	4482	CHS			
			Angina (Y/N)			2.10e-4	0.25(0.07)	13308	WHI			
			Ever had pain/discomfort in your chest (Y/N)			1.06e-4	0.21(0.05)	4477	CHS			
CDKN2A, CDKN2B	RS1333049	C (0.5)	Chest pain or discomfort? (Y/N)	ANGINA	EA	4.69e-3	0.08(0.03)	9338	ARIC	3	Coronary Artery Disease, Type 2 Diabetes, Hypertension	17554300, 17634449
			See a doctor because of chest pain? (Y/N)			8.80e-3	0.32(0.12)	572	ARIC			
			Ever had pain/discomfort in your chest (Y/N)			9.72e-3	0.11(0.04)	4472	CHS			
			Angina (Y/N)			7.75e-3	0.14(0.05)	13331	WHI			
LPL	RS6586891	A (0.6)	LN1 Total triglycerides in mmol/l	Serum Triglycerides	EA	7.76e-7	−0.02(4.75e-3)	9326	ARIC	3	HDL Cholesterol	18193043
			Triglyceride (mg/dl)			3.45e-3	−5.18(1.77)	4469	CHS			
			LN1 core triglyceride (mg/dl)			6.68e-3	−0.06(0.02)	922	WHI			
			Total Triglycerides (mmol/l)			3.50e-4	−0.06(0.02)	9326	ARIC			
			LN1 Triglycerides (mg/dl)			2.45e-3	−0.03(9.65e-3)	4469	CHS			
CDKN2A, CDKN2B	RS10757278	A (0.5)	Ever had pain/discomfort in your chest (Y/N)	ANGINA	EA	5.96e-3	−0.12(0.04)	4474	CHS	3	Myocardial infarction	17478679
			Angina (Y/N)			6.59e-3	−0.14(0.05)	13313	WHI			
			Chest pain or discomfort? (Y/N)			9.73e-3	−0.07(0.03)	11047	ARIC			
			See a doc because of chest pain? (Y/N)			4.44e-3	−0.31(0.11),	722	ARIC			
			Rose angina (Y/N)			8.17e-3	0.16(0.06)	11040	ARIC			
LDLR	RS6511720	G (0.9)	Total cholesterol (mmol/l)	Serum Cholesterol	EA	6.84e-16	0.18(0.02)	10891	ARIC	2	LDL Cholesterol	18193043
			Total cholesterol (mg/dl)			2.64e-5	12.05(2.85)	924	WHI			
			LN1 Total cholesterol (mmol/l)			4.15e-17	0.03(3.30e-3)	10891	ARIC			
			High cholesterol requiring pills ever (Y/N)			10.00e-12	0.42(0.06)	12431	WHI			
			LN1 Total cholesterol (mg/dl)			1.87e-5	0.05(0.01)	924	WHI			
PCSK9	RS11591147	G (0.99)	Total cholesterol (mmol/l)	Serum Cholesterol	EA	1.13e-16	0.46(0.06)	10833	ARIC	2	LDL Cholesterol, lipids	18193044
			Total cholesterol (mg/dl)			8.47e-3	18.62(7.06)	924	WHI			
			LN1 total cholesterol (mg/dl)			4.67e-3	0.09(0.03)	924	WHI			
			High cholesterol requiring pills ever (Y/N)			4.72e-6	0.92(0.20)	12419	WHI			
			LN1 total cholesterol (mmol/l)			1.59e-18	0.07(8.39e-3)	10833	ARIC			
APOE, APOC1, APOC4, APOC2	RS4420638	A (0.8)	High cholesterol requiring pills ever (Y/N)	Serum Cholesterol	EA	1.18e-6	−0.22(0.04)	12430	WHI	2	LDL Cholesterol, lipids, Alzheimer's disease, Coronary Artery Disease, C-reactive protein, Sporadic late onset Alzheimer's disease, ApoB, Triglycerides, Total cholesterol	17474819, 17463246, 18193043, 18802019, 17998437, 19197348, 19567438
			Total cholesterol (mmol/l)			2.31e-12	−0.14(0.02)	9323	ARIC			
			LN1 Total cholesterol (mmol/l)			1.96e-13	−0.02(3.00e-3)	9323	ARIC			
			High cholesterol requiring pills ever (Y/N)			1.18e-6	−0.22(0.04)	12430	WHI			
			Total cholesterol (mmol/l)			2.31e-12	−0.14(0.02)	9323	ARIC			
			LN1 Total cholesterol (mmol/l)			1.96e-13	−0.02(3.00e-3)	9323	ARIC			
												
TMEM18	RS6548238	C (0.8)	Hip circumference cm	Hip Circumference	EA	5.99e-3	0.62(0.22)	4456	CHS	2	Body Mass Index	19079261
			Hip circumference cm			7.12e-3	0.59(0.22)	13272	WHI			
			LN1 hip circumference cm			6.85e-3	5.84e-3(2.16e-3)	4456	CHS			
			LN1 hip circumference cm			3.93e-3	5.60e-3(1.94e-3)	13272	WHI			
TMEM18	RS6548238	C (0.9)	High blood pressure ever diagnosed?	Hypertension	AA	7.69e-3	0.19(0.07)	3946	ARIC	2	Body Mass Index	19079261
			Pills for hypertension now (Y/N)			7.17e-3	0.18(0.07)	4122	WHI			
			Hypertension category: never hypertensive (Y/N)			1.03e-3	−0.22(0.07)	4076	WHI			
			Hypertension ever (Y/N)			5.85e-4	0.23(0.07)	4206	WHI			
			Pills for hypertension ever (Y/N)			6.30e-3	0.19(0.07)	4186	WHI			
			Hypertension category: treated hypertensive (Y/N)			6.54e-3	0.19(0.07)	4076	WHI			
FTO	RS3751812	T (0.4)	Waist circumference (cm)	WaistSize	EA	3.88e-3	1.33(0.46)	2360	EAGLEIII	2	Body Mass Index	17658951
			LN1 waist circumference cm			3.68e-8	0.01(1.99e-3)	13282	WHI			
			LN1 waist circumference (cm)			2.73e-3	0.01(4.99e-3)	2360	EAGLEIII			
			Waist circumference cm			2.32e-8	1.03(0.18)	13282	WHI			
CELSR2, PSRC1	RS599839	A (0.3)	Re-calibrated HDL(3) cholesterol in mg/dl	HDL	AA	3.07e-3	−1.00(0.34)	3023	ARIC	2	Coronary Artery Disease, LDL cholesterol, Total cholesterol	18262040, 18193043, 18179892, 17634449, 18193044
			LN1 high-density lipoprotein-3 (mg/dl)			2.55e-3	−0.03(9.54e-3)	974	WHI			
			LN1 HDL Cholesterol in mg/dl			4.60e-3	−0.02(8.74e-3)	3024	ARIC			
			High-density lipoprotein-2 (mg/dl)			2.94e-3	−1.09(0.36)	974	WHI			
			LN1 re-calibrated HDL(3) cholesterol in mg/dl			2.06e-3	−0.03(9.10e-3)	3023	ARIC			
			HDL cholesterol (mg/dl)			1.51e-3	−2.20(0.69)	982	WHI			
			LN1 high-density lipoprotein-2 (mg/dl)			2.43e-3	−0.06(0.02)	974	WHI			
			LN1 HDL cholesterol (mg/dl)			1.23e-3	−0.04(0.01)	982	WHI			
			High-density lipoprotein-3 (mg/dl)			2.70e-3	−1.20(0.40)	974	WHI			
CDKN2A, CDKN2	RS1333049	C (0.5)	Percutaneous transluminal coronary angioplasty (Y/N)	Artery Treatment	EA	2.93e-6	0.17(0.04)	13331	WHI	2	Coronary Artery Disease, Type 2 Diabetes, Hypertension	17554300, 17634449
			Coronary bypass surgery (Y/N)			6.61e-3	0.28(0.10)	4446	CHS			
CDKN2A, CDKN2B	RS1333049	C (0.5)	Myocardial infarction status at baseline (Y/N)	Cardiac	EA	8.24e-3	0.17(0.06)	4477	CHS	2	Coronary Artery Disease, Type 2 Diabetes, Hypertension	17554300, 17634449
			Myocardial infarction (Incident or Prevalent))			2.03e-3	0.22(0.07)	4477	CHS			
			Myocardial infarction (Y/N)			8.78e-5	0.11(0.03)	13331	WHI			
APOA1, APOC3, APOA4, APOA5	RS964184	G (0.3)	LN1 serum cholesterol (mg/dl)	Serum Cholesterol	MA	2.18e-3	0.02(7.61e-3),	2034	EAGLEIII	2	HDL Cholesterol, lipids, Triglycerides, Triglyceride levels	18193043
			LN1 serum cholesterol (mmol/l)			2.91e-3	0.02(6.36e-3)	2040	EAGLEIII			
			Serum cholesterol (mmol/l)			2.50e-3	0.12(0.04)	2034	EAGLEIII			
			High cholesterol requiring pills ever (Y/N)			3.39e-3	0.29(0.10)	1859	WHI			
			Serum cholesterol (mmol/l)			3.44e-3	0.12(0.04)	2040	EAGLEIII			
			LN1 serum cholesterol (mg/dl)			2.98e-3	0.02(7.64e-3)	2040	EAGLEIII			
			Serum cholesterol (mg/dl)			3.42e-3	4.50(1.53)	2040	EAGLEIII			
			LN1 serum cholesterol: si (mmol/l)			2.10e-3	0.02(6.35e-3)	2034	EAGLEIII			
			Serum cholesterol (mg/dl)			2.50e-3	4.64(1.53)	2034	EAGLEIII			
APOA1, APOC3, APOA4, APOA5	RS964184	G (0.1)	LN1 total cholesterol (mg/dl)	Serum Cholesterol	EA	1.53e-3	0.03(0.01)	924	WHI	2	HDL Cholesterol, lipids, Triglycerides, Triglyceride levels	18193043
			Total cholesterol (mg/dl)			3.02e-3	7.46(2.51)	924	WHI			
			Serum cholesterol (mmol/l)			8.29e-3	0.13(0.05)	2592	EAGLEIII			
			High cholesterol requiring pills ever (Y/N)			2.06e-14	0.35(0.05)	12433	WHI			
			Serum cholesterol (mg/dl)			8.28e-3	4.89(1.85)	2592	EAGLEIII			
TIMD4, HAVCR1	RS1501908	C (0.6)	LN1 serum cholesterol (mmol/l)	Serum Cholesterol	EA	9.63e-3	0.01(5.48e-3)	2582	EAGLEIII	2	LDL Cholesterol	19060906
			LN1 total cholesterol in mmol/l			4.04e-4	8.64e-3(2.44e-3)	9323	ARIC			
			Serum cholesterol (mmol/l)			9.35e-3	0.09(0.03)	2584	EAGLEIII			
			Serum cholesterol (mg/dl)			9.40e-3	3.45(1.33)	2584	EAGLEIII			
			Total cholesterol in mmol/l			7.27e-4	0.05(0.02)	9323	ARIC			
			LN1 serum cholesterol (mmol/l)			8.93e-3	0.01(5.43e-3)	2584	EAGLEIII			
			LN1 serum cholesterol (mg/dl)			9.83e-3	0.02(6.52e-3)	2582	EAGLEIII			
			LN1 serum cholesterol (mg/dl)			9.17e-3	0.02(6.49e-3)	2584	EAGLEIII			
LDLR	RS6511720	G (0.9)	Total cholesterol in mmol/l	Serum Cholesterol	AA	1.50e-10	0.25(0.04)	3786	ARIC	2	LDL Cholesterol	18193043
			LN1 total cholesterol (mg/dl)			8.37e-4	0.04(0.01)	1009	WHI			
			High cholesterol requiring pills ever (Y/N)			2.21e-3	0.31(0.10)	4041	WHI			
			Total cholesterol (mg/dl)			1.36e-3	8.31(2.59)	1009	WHI			
			LN1 total cholesterol in mmol/l			6.45e-11	0.04(5.85e-3)	3786	ARIC			
ABCG8	RS6544713	C (0.7)	High cholesterol requiring pills ever (Y/N)	Serum Cholesterol	EA	7.94e-5	−0.14(0.04)	12438	WHI	2	LDL Cholesterol	19060906
			Total cholesterol in mmol/l			2.23e-9	−0.09(0.02)	10826	ARIC			
			LN1 total cholesterol in mmol/l			2.13e-9	−0.01(2.34e-3)	10826	ARIC			
APOB	RS754523	T (0.7)	LN1 total cholesterol in mmol/l	Serum Cholesterol	EA	3.01e-10	−0.02(2.49e-3)	9314	ARIC	2	LDL Cholesterol	19750184
			High cholesterol requiring pills ever (Y/N)			4.76e-4	−0.13(0.04)	12427	WHI			
			Total cholesterol in mmol/l			5.52e-10	−0.10(0.02)	9314	ARIC			
APOE, APOC1, APOC4, APOC2	RS4420638	A (0.8)	LN1 dietary cholesterol (mg) (dietary consumption)	Cholesterol MG	EA	1.48e-3	0.03(9.34e-3)	9194	ARIC	2	LDL Cholesterol, lipids, Alzheimer's disease, Coronary Artery Disease, C-reactive protein, Sporadic late onset Alzheimer's disease, ApoB, Triglycerides, Total cholesterol	17474819, 17463246, 18193043, 18802019, 17998437, 19197348, 19567438
			Dietary cholesterol (mg)			9.48e-3	5.84(2.25)	13291	WHI			
			LN1 dietary cholesterol (mg)			4.35e-3	0.03(9.66e-3)	13291	WHI			
			Dietary cholesterol (mg)			3.28e-3	6.94(2.36)	9194	ARIC			
CDKN2A, CDKN2B	RS10757278	A (0.5)	Coronary bypass surgery (Y/N)	Artery Treatment	EA	9.60e-3	−0.26(0.10)	4448	CHS	2	Myocardial infarction	17478679
			Percutaneous transluminal coronary angioplasty (Y/N)			2.86e-6	−0.17(0.04)	13313	WHI			
FTO	RS8050136	A (0.4)	LN1 hip circumference cm	Hip Circumference	EA	3.11e-7	7.67e-3(1.50e-3)	13272	WHI	2	Obesity, Type 2 diabetes	17554300, 19079260, 17463249, 17463248, 18372903, 18159244, 19056611, 17658951
			LN1 hip girth to nearest cm			1.26e-8	7.35e-3(1.29e-3)	9334	ARIC			
			Hip girth to nearest cm			7.26e-9	0.81(0.14)	9334	ARIC			
			Hip circumference cm			6.06e-7	0.84(0.17)	13272	WHI			
FTO	RS8050136	A (0.4)	LN1 waist hip ratio	Waist Hip Ratio	EA	7.15e-3	1.46e-3(5.43e-4)	13269	WHI	2	Obesity, Type 2 diabetes	17554300, 19079260, 17463249, 17463248, 18372903, 18159244, 19056611, 17658951
			Waist-to-hip ratio			1.71e-7	6.11e-3(1.17e-3)	9333	ARIC			
			LN1 waist-to-hip ratio			1.75e-7	3.21e-3(6.10e-4)	9333	ARIC			
FTO	RS8050136	A (0.4)	LN1 waist circumference cm	Waist Size	EA	2.88e-8	0.01(1.99e-3)	13284	WHI	2	Obesity, Type 2 diabetes	17554300, 19079260, 17463249, 17463248, 18159244, 19056611, 17658951
			LN1 waist girth to nearest cm			3.63e-12	0.01(2.01e-3)	9334,9334	ARIC			
			Waist girth to nearest cm			1.29e-12	1.40(0.20)	13284	ARIC			
			Waist circumference cm			1.91e-8	1.03(0.18)		WHI			
IGF2BP2	RS4402960	G (0.7)	LN1 circulating glucose value in mg/dl	Plasma Serum Glucose	EA	7.38e-3	−8.10e-3(3.02e-3)	9339	ARIC	2	Type 2 Diabetes	17463249, 17463248, 17463246, 19401414
			Baseline glucose (mg/dl)			4.80e-3	−2.07(0.73)	4468	CHS			
			LN1 baseline glucose (mg/dl)			2.28e-3	−0.01(4.84e-3)	4468	CHS			
TCF7L2	RS7903146	C (0.7)	LN1 circulating glucose (mg/dl)	Plasma Serum Glucose	EA	8.39e-3	−0.02(8.95e-3)	918	WHI	2	Type 2 Diabetes	19734900, 17668382, 17463246, 17463248, 18372903, 17293876, 17460697, 19056611, 19401414
			LN1 circulating glucose value in mg/dl			2.09e-7	−0.02(2.99e-3)	10739	ARIC			
			Circulating glucose value in mg/dl			1.73e-5	−2.04(0.48)	10739	ARIC			

Related associations that met the criteria for PheWAS significance are given here, sorted by the number of PAGE site replications for a given phenotype-class. Related associations were defined as SNPs significantly associated in this PheWAS with phenotype-classes closely related to phenotypes among known associations. Significance was defined as a test of association with p<0.01 observed in two or more PAGE studies for the same SNP, phenotype-class, and race/ethnicity and consistent direction of effect when relevant. For each, the nearest gene(s), the SNP rs number, coded allele (CA) and frequency (CAF), associated phenotypes, phenotype-class, race/ethnicity, p-values, genetic effect/beta values (standard error; SE), sample sizes, substudies, number of substudies with results passing our p-value cutoff, the previously associated phenotype for that SNP, and references for the previously associated phenotypes are given.

1 Coded Allele.

2 Coded allele frequency.

3 Associated phenotypes for individual results.

4 Phenotype-class.

5 Race/ethnicity for association, abbreviations: African American (AA), European American (EA), Mexican American/Hispanic (H).

6 P-Values of results that passed p = 0.01 threshold in order of the associated phenotypes.

7 Beta and standard error in order of the associated phenotypes.

8 Sample size in order of the associated phenotypes.

9 Studies with the significant result, in order of the associated phenotypes.

10 Total number of studies with at least one result passing p-value threshold for specific phenotype-class and SNP.

11 Previously reported associated phenotypes for SNP.

12 Pubmed ID's for previously associated phenotypes.

**Table 4:** PheWAS Tests of Association: Novel Associations.

Nearest Gene	SNP ID	CA^1^ (CAF^2^)	Phenotypes^8^	Associated Phenotype Class^3^	Ethnicity^5^	P-Values^6^	Beta (SE)^7^	Sample Size^9^	Substudies^10^	Substudy Count^11^	Previously Associated Phenotype^4^	References^12^
CELSR2,PSRC1	RS599839	A (0.3)	White blood cell (kcell/ml)	White Blood Count	AA	2.14e-3	0.88(0.29)	4147	WHI	3	Coronary Artery Disease, LDL cholesterol, Total cholesterol	18262040, 18193043, 18179892, 17634449, 18193044
			White blood count			3.69e-7	0.29(0.06)	3049	ARIC			
			LN1 white blood count (×1,000/cubic mm)			5.62e-4	0.05(0.01)	777	CHS			
			LN1 white blood cell (kcell/ml)			9.99e-15	0.05(6.03e-3)	4147	WHI			
			LN1 white blood count			1.28e-8	0.04(7.65e-3)	3049	ARIC			
NOTCH2	RS10923931	G (0.7)	LN1 white blood cell count	White Blood Count	AA	9.59e-4	0.03(8.51e-3)	2083	EAGLEIII	3	Type 2 Diabetes, Type I Diabetes	18372903
			LN1 white blood cell count			9.59e-4	0.03(8.51e-3)	2083	EAGLEIII			
			White blood count			8.74e-4	0.18(0.05)	3051	ARIC			
			White blood cell count			2.75e-4	0.25(0.07)	2083	EAGLEIII			
			LN1 white blood cell (kcell/ml)			1.36e-10	0.04(6.14e-3)	4215	WHI			
			White blood cell count			2.75e-4	0.25(0.07)	2083	EAGLEIII			
			LN1 white blood count			1.25e-3	0.02(7.14e-3)	3051	ARIC			
IL6R	RS2228145	A (0.9)	White blood count	White Blood Count	AA	4.17e-8	−0.36(0.06)	3806	ARIC	3	C-reactive Protein	20186139
			White blood cell count			2.83e-4	−0.35(0.10)	2038	EAGLEIII			
			LN1 white blood count (×1,000/cubic mm)			5.61e-4	−0.06(0.02)	783	CHS			
			White blood cell count			2.83e-4	−0.35(0.10)	2038	EAGLEIII			
			LN1 white blood count			8.42e-10	−0.05(8.70e-3)	3806	ARIC			
			LN1 white blood cell count			3.57e-4	−0.04(0.01)	2038	EAGLEIII			
			LN1 white blood cell count			3.57e-4	−0.04(0.01)	2038	EAGLEIII			
NEGR1	RS2815752	T (0.08)	Ever smoked cigarettes (Y/N)	Ever Smoked	EA	7.57e-3	0.08(0.03)	9339	ARIC,WHI	2	Body Mass Index	19079261
			Smoked at least 100 cigarettes ever (Y/N)			7.48e-4	0.09(0.03)	13222				
IL6	RS1800795	C (0.06)	LN1 beta carotene mcg (dietary consumption)	Carotene	AA	5.43e-3	0.12(0.04)	3071	ARIC	2	C-reactive Protein	15820616, 16544245, 16644865, 17003362, 17416766, 17623760, 17694420, 17916900, 17996468, 18041006, 18239642, 18257935, 18276608, 18321738, 18449426, 18458677, 18752089, 18992263, 19056105, 19106168, 19140096, 19267250, 19280716, 19330901, 19377912, 19387461, 19435922, 19452524, 19542902, 19592000, 19671870, 19833146, 19853505, 19876004, 20043205, 20044998, 20132806, 20149313, 20175976, 20176930, 20333461, 20361391, 20436380, 20459474, 20592333, 20622166
			LN1 carotenes (dietary consumption)			9.44e-3	0.24(0.09)	1964	EAGLEIII			
			LN1 alpha carotene mcg (dietary consumption)			2.74e-3	0.26(0.09)	3071	ARIC			
IL6R	RS2228145	A (0.9)	LN1 lymphocytes	Lymphocytes	AA	5.12e-10	0.06(0.01)	3769	ARIC	2	C-reactive Protein	20186139
			Lymphocytes (percent of 100 cells)			5.39e-5	2.96(0.73)	949	EAGLEIII			
			Lymphocytes			6.63e-10	2.35(0.38)	3769	ARIC			
			LN1 lymphocytes (percent of 100 cells)			3.88e-5	0.08(0.02)	949	EAGLEIII			
IL6R	RS2228145	A (0.9)	LN1 neutrophils	Neutrophils	AA	1.54e-8	−0.06(9.81e-3)	3769	ARIC	2	C-reactive Protein	20186139
			LN1 Segmented neutrophils			2.44e-4	−0.06(0.02)	949	EAGLEIII			
			Neutrophils			4.66e-10	−2.61(0.42)	3769	ARIC			
			Segmented neutrophils			7.74e-5	−3.12(0.79)	949	EAGLEIII			
CELSR2,PSRC1	RS599839	A (0.8)	Currently of on a special diet? (Y/N)	Dieting	EA	9.48e-3	0.12(0.05)	9328	ARIC	2	Coronary Artery Disease, LDL cholesterol, Total cholesterol	18262040, 18193043, 18179892, 17634449, 18193044
			Low-fat or low cholesterol diet (Y/N)			5.87e-3	0.08(0.03)	13068	WHI			
GALNT2	RS2144300	C (0.8)	LN1 FEV(3) over FEV(6) (Lung function)	FEV3	AA	4.90e-4	−0.11(0.03)	3090	ARIC	2	Coronary Heart Disease, HDL cholesterol, Triglycerides	18193043
			FEV(3) (liters) (Lung function)			1.13e-3	−0.10(0.03)	3090	ARIC			
			LN1 FEV3 at 3.0 seconds Largest value (Lung Function)			8.82e-3	−0.03(0.01)	1953	ARIC			
			FEV(3) over FEV(6) (Lung Function)			7.93e-4	−2.26(0.67)	3090	EAGLEIII			
			LN1 FEV(3) (liters) (Lung Function)			8.43e-4	−0.03(7.92e-3)	3090	ARIC			
GALNT2	RS2144300	C (0.8)	Pills for hypertension ever (Y/N)	Hypertension	AA	8.27e-3	0.15(0.06)	4180	WHI	2	Coronary Heart Disease, HDL cholesterol, Triglycerides	18193043
			High blood pressure ever diagnosed? (Y/N)			1.61e-3	0.24(0.08)	3142	ARIC			
GALNT2	RS2144300	C (0.4)	LN1 serum total calcium: (mmol/l)	Serum Calcium	EA	8.10e-3	−2.36e-3(8.92e-4)	2587	EAGLEIII	2	Coronary Heart Disease, HDL cholesterol, Triglycerides	18193043
			LN1 serum calcium (mg-dl)			1.47e-3	−1.84e-3(5.80e-4)	9098	ARIC			
			Serum total calcium			5.95e-3	0.03(0.01)	2585	EAGLEIII			
			Serum total calcium (mmol/l)			7.47e-3	−7.97e-3(2.98e-3)	2587	EAGLEIII			
			LN1 serum total calcium (mmol/l)			6.28e-3	−2.39e-3(8.75e-4)	2585	EAGLEIII			
			LN1 serum total calcium			6.40e-3	−3.09e-3(1.13e-3)	2585	EAGLEIII			
			Serum calcium (mg-dl)			1.71e-3	−0.02(6.24e-3)	9098	ARIC			
			Serum total calcium (mmol/l)			5.95e-3	−7.98e-3(2.90e-3)	2585	EAGLEIII			
GALNT2	RS2144300	C (0.8)	LN1 white blood cell (kcell/ml)	White Blood Count	AA	3.32e-6	−0.04(7.53e-3)	4210	WHI	2	Coronary Heart Disease, HDL cholesterol, Triglycerides	18193043
			LN1 white blood count (×1,000/cubic mm)			7.96e-3	−0.05(0.02)	785	CHS			
GALNT2	RS2144300	C (0.4)	Coronary artery bypass graft (cabg) (Y/N)	Artery Treatment	EA	2.46e-3	0.24(0.08)	13152	WHI	2	Coronary Heart Disease, HDL cholesterol, Triglycerides	18193043
			Aortic Aneurysm repair (Y/N)			5.49e-3	0.57(0.20)	4250	CHS			
LIPG	RS2156552	A (0.2)	Doctor ever told you had goiter? (Y/N)	Thyroid Goiter	EA	8.60e-3	−1.91(0.25)	2273	EAGLEIII	2	HDL Cholesterol	18193043
			Age told had goiter/thyroid disease			7.57e-3	−2.97(1.11)	763	ARIC			
CETP	RS3764261	T (0.3)	Age at first period category: 11 years	Menstruation	EA	4.30e-3	−0.11(0.04)	13276	WHI	2	HDL Cholesterol, LDL cholesterol, Waist circumference	18193043, 19359809
			Age when menstruation began			2.04e-3	0.11(0.03)	5705	WHI			
			LN1 age when menstruation began			5.43e-4	9.19e-3(2.66e-3)	5705	ARIC			
			Age at first period category: 10 years			6.81e-3	−0.17(0.06)	13276	ARIC			
FADS1, FADS2, FADS3	RS174547	C (0.3)	Platelet count	Platelet Count	EA	7.37e-3	3.26(1.22)	9174	ARIC	2	HDL Cholesterol, Triglycerides, Total cholesterol, LDL cholesterol	20037589, 19060906
			LN1 platelet count (kcell/ml)			2.28e-3	0.01(3.30e-3)	13140	WHI			
			LN1 platelet count			1.68e-3	0.01(3.90e-3)	9174	ARIC			
LDLR	RS6511720	G (0.9)	Max % arterial stenosis (1–24%) (Arterial measurement)	Artery	EA	4.19e-3	−0.22(0.08)	4460	CHS	2	LDL Cholesterol	18193043
			Arterial plaque in any site (Y/N)			9.19e-3	0.13(0.05)	8269	ARIC			
			Max % arterial stenosis (25–49%) (Arterial measurement)			4.91e-3	0.21(0.07)	4460	CHS			
PCSK9	RS11591147	G (0.996)	Coronary artery bypass graft (Y/N)	Artery Treatment	AA	2.11e-4	−2.23(0.60)	4274	WHI	2	LDL Cholesterol, lipids	18193044
			Heart or arterial surgery? (Y/N)			1.53e-3	−1.97(0.62)	3909	ARIC			
APOE, APOC1, APOC4, APOC2	RS4420638	A (0.8)	Circulating glucose value in mg/dl	Plasma Serum Glucose	EA	8.86e-4	1.89(0.57)	9336	ARIC	2	LDL Cholesterol, lipids, Alzheimer's disease, Coronary Artery Disease, C-reactive protein, Sporadic late onset Alzheimer's disease, ApoB, Triglycerides, Total cholesterol	17474819, 17463246, 18193043, 18802019, 17998437, 19197348, 19567438
			Baseline glucose (mg/dl)			4.65e-3	2.67(0.94)	4470	CHS			
			LN1 circulating baseline glucose (mg/dl)			5.67e-3	0.02(6.23e-3)	4470	CHS			
			LN1 circulating glucose value in mg/dl			6.81e-4	0.01(3.62e-3)	9336	ARIC			
CELSR2, PSRC1,SORT1	RS646776	G (imputed)	Are you currently on a special diet (Y/N)	Dieting	EA	4.20e-3	−0.14(0.05)	9331	ARIC	2	LDL Cholesterol, Myocardial infarction (early onset)	18193044, 18262040
			Low-fat or low cholesterol diet (Y/N)			8.78e-3	−0.08(0.03)	13051	WHI			
APOB	RS562338	T (0.6)	Are you following a special diet (Y/N)	Dieting	AA	4.82e-3	−0.34(0.12)	817	CHS	2	LDL Cholesterol, Total cholesterol, Type 2 Diabetes	18193043
			Currently of diet (Y/N)			5.07e-3	−0.19(0.07)	3149	ARIC			
CDKN2A,CDKN2B	RS10757278	A (0.8)	LN1 white blood count	White Blood Count	AA	5.05e-4	−0.03(7.32e-3)	3877	ARIC	2	Myocardial infarction	17478679
			White blood count			3.24e-3	−0.16(0.05)	3877	ARIC			
			LN1 white blood cell (kcell/ml)			3.80e-3	−0.02(6.96e-3)	4214	WHI			
CDKN2A,CDKN2B	RS2383207	A (imputed)	LN1 dietary vitamin b12 (mcg)	VitaminB12	EA	4.34e-3	0.02(5.69e-3)	13254	WHI	2	Myocardial infarction	17478679
			Dietary vitamin b12 (mcg)			3.35e-3	0.14(0.05)	13254	WHI			
			LN1 vitamin b12 (micrograms)			3.95e-3	0.02(6.98e-3)	9197	ARIC			
ANGPTL3	RS1748195	G (0.6)	LN1 hemoglobin (g/dl)	Hemoglobin	AA	7.66e-3	−9.61e-3(3.60e-3)	2085	EAGLEIII	2	Triglycerides, lipids	18193043
			LN1 hemoglobin (gm/dl)			9.57e-3	−4.64e-3(1.79e-3)	4195	WHI			
			Hemoglobin (g/dl)			6.28e-3	−0.14(0.05)	2085	EAGLEIII			
ADAMTS9	RS4607103	C (0.8)	LN1 min lumen diameter (arterial measurement)	Artery	EA	7.00e-3	0.07(0.03)	120	ARIC	2	Type 2 Diabetes	18372903
			LN1 average near and far wall max common carotid artery (mm) (Arterial measurement)			6.22e-3	6.32e-3(2.31e-3)	4460	CHS			
			Ave near and far wall max common carotid artery (mm) (Arterial measurement)			7.91e-3	0.01(5.02e-3)	4460	CHS			
ADAMTS9	RS4607103	C (0.8)	Smoked at least 100 cigarettes ever? (Y/N)	Ever Smoked	EA	1.38e-3	−0.09(0.03)	13214	WHI	2	Type 2 Diabetes	18372903
			Ever smoked? (Y/N)			4.11e-3	−0.14(0.05)	4477	CHS			
IGF2BP2	RS4402960	G (0.7)	Currently of diet? (Y/N)	Dieting	EA	3.14e-4	−0.15(0.04)	9331	ARIC	2	Type 2 Diabetes	17463249, 17463248, 17463246, 19401414
			Diabetic or ADA diet? (Y/N)			9.63e-4	−0.23(0.07)	12958	WHI			
TCF7L2	RS7901695	C (0.5)	LN1 white blood count, white blood count	White Blood Count	AA	2.75e-3	−0.02(6.77e-3)	3053	ARIC	2	Type 2 Diabetes	17554300, 17463249, 17463246, 17668382
			LN1 white blood cell (kcell/ml)			7.03e-3	−0.14(0.05)	3053	ARIC			
						7.73e-3	−0.02(5.76e-3)	4182	WHI			
TSPAN8/LGR5	RS7961581	C (0.3)	Pulse rate (beats/min) (age 5+ years)	HeartRate	EA	8.38e-3	−1.33(0.50)	2570	EAGLEIII	2	Type 2 Diabetes	18372903
			LN1 heart rate per minute			8.17e-3	−6.20e-3(2.34e-3)	9314	ARIC			
NOTCH2	RS10923931	G (0.7)	Hypertension ever (Y/N)	Hypertension	AA	3.07e-3	−0.14(0.05)	4206	WHI	2	Type 2 Diabetes, Type I Diabetes	18372903
			History of high blood pressure from baseline questionnaire (Y/N)			2.00e-3	−0.14(0.04)	4736	MEC			
			History of high blood pressure (Y/N)			3.50e-3	−0.14(0.05)	4362	MEC,			
			Hypertension interested category: never Hypertensive (Y/N)			2.33e-3	−0.15(0.05)	4076	WHI			
			Hypertension interested category: treated Hypertensive (Y/N)			2.92e-3	−0.14(0.05)	4076	WHI			
			Pills for hypertension ever (Y/N)			8.18e-4	−0.16(0.05)	4186	WHI			
			Pills for hypertension now (Y/N)			2.82e-3	−0.14(0.05)	4122	WHI			
FTO	RS8050136	A (0.4)	Age at first period interested category: 14 years	Menstruation	EA	5.26e-3	−0.10(0.04)	13275	WHI	2	Obesity, Type 2 diabetes	17554300, 19079260, 17463249, 17463248, 18159244, 19056611, 17658951
			Age when menstruation began			1.18e-4	−0.14(0.04)	4917	ARIC			
			LN1 age when Menstruation began			6.09e-5	−0.01(2.74e-3)	4917	ARIC			
CELSR2, PSRC1, SORT1	RS646776	G (imputed)	Hospitalized for chest pain? (Y/N)	ANGINA	EA	3.05e-3	−0.55(0.18)	568	ARIC	2	LDL Cholesterol	19060911, 19198609, 18193044, 18262040, 19060910
			Angina (Y/N)			3.17e-4	−0.25(0.07)	13289	WHI			
CDKN2A,CDKN2B	RS1333049	C (0.2)	Hemoglobin (g/dl)	Hemoglobin	AA	5.40e-3	0.15(0.05)	2092	EAGLEIII	2	Coronary Artery Disease, Type 2 Diabetes, Hypertension	17554300, 17634449
			Hemoglobin (g/dl)			8.21e-3	0.26(0.10)	786	CHS			
			LN1 Hemoglobin (g/dl)			4.07e-3	0.01(3.95e-3)	2092	EAGLEIII			

Novel associations that met the criteria for PheWAS significance are given here, sorted by the most to least number of PAGE study sites available. Related associations were defined as SNPs significantly associated in this PheWAS with phenotype-classes closely related to phenotypes among known associations. Significance was defined as a test of association with p<0.01 observed in two or more PAGE studies for the same SNP, phenotype class, and race/ethnicity and consistent direction of effect when relevant. For each, the nearest gene(s), the SNP rs number, coded allele (CA) and frequency (CAF), associated phenotypes, phenotype-class, race/ethnicity, p-values, genetic effect/beta values (standard error; SE), sample sizes, substudies, number of substudies with results passing our p-value cutoff, the previously associated phenotype for that SNP, and references for the previously associated phenotypes are given.

1 Coded Allele.

2 Coded allele frequency.

3 Associated phenotypes.

4 Phenotype-class.

5 Race/ethnicity for association, abbreviations: African American (AA), European American (EA), Mexican American/Hispanic (H).

6 P-Values of results that passed p = 0.01 threshold in order of the associated phenotypes.

7 Beta and standard error in order of the associated phenotypes.

8 Sample size in order of the associated phenotypes.

9 Studies with the significant result, in order of the associated phenotypes.

10 Total number of studies with at least one result passing p-value threshold for specific phenotype class and SNP.

11 Previously reported associated phenotypes for the SNP.

12 Pubmed ID's for previously associated phenotypes.

### Known Associations—Validating the PheWAS Approach

**Figure 1:**
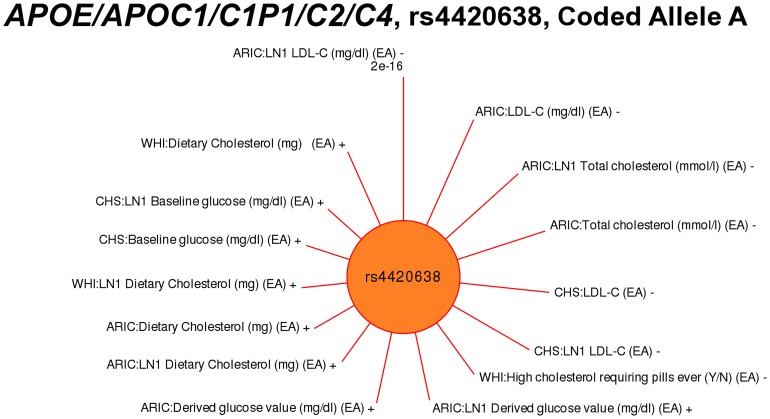
PheWAS associations for rs4420638 near *APOC1*. SNP rs4420638 has previously been associated with LDL cholesterol levels, triglycerides, Alzheimer's disease, coronary artery disease, and sporadic late onset Alzheimer's. The length of the lines correspond to –log10(p-value), and the lines are plotted clockwise starting at top for the association with the smallest p-value. Lines are labeled with the study-specific phenotype, the PAGE study, racial/ethnic group, and direction of effect (+ or −). Red lines represent associations at p<0.01. “LN1” indicates the phenotype had 1 added to the variable, and then the variable was natural log transformed. The PheWAS phenotypes significantly associated with this SNP varied, with known associations for LDL cholesterol levels, as well as the related phenotypes “Total cholesterol (mmol/l)” and “Dietary cholesterol (mg)”, and novel phenotypes such as “Baseline glucose (mg/dl)”.

Almost half of the PAGE PheWAS results (52/111; 48%) replicated previously known genotype-phenotype associations. These replicated results serve as positive controls and demonstrate that the high-throughput PheWAS approach is feasible and valid. As an example, low-density lipoprotein cholesterol (LDL-C) has previously been associated with rs4420638 near *APOE/APOC1/C1P1/C2/C4* in European Americans [8], [9]. In the PAGE PheWAS, a significant association between the same SNP and LDL-C phenotypes of the “LDL-C” phenotype-class in European Americans as reported in the literature [8], [9] was observed in two PAGE study sites, with the same direction of effect (β) as well as a third PAGE site with near significant results: ARIC (p = 1.27×10^−15^, β = −5.75), CHS (p = 7.89×10^−12^, β = −7.06), and WHI (p = 0.06, β = −4.15). Figure 1 shows the significant PheWAS LDL-C results, as well as other associations considered significant for rs4420638 across PAGE study sites for other phenotype-classes in a similar racial/ethnic group passed a significance threshold of p<0.01 with a consistent direction of genetic effect.

### Related Associations

**Figure 2:**
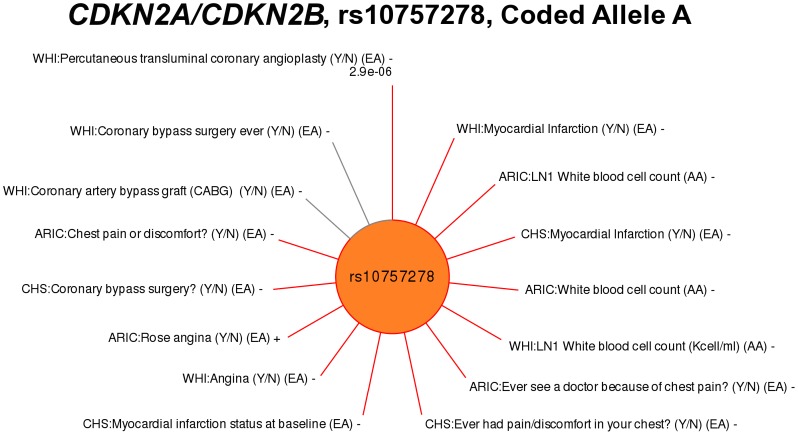
PheWAS associations for rs10757278 near *CDKN2A/CDKN2B*. SNP rs10757278 was previously associated with myocardial infarction (MI). Associations are plotted clockwise starting at top for the association with the smallest p-value and the length of the line corresponds to –log10(p-value). Lines are labeled with the study-specific phenotype, the PAGE study, racial/ethnic group, and direction of effect (+ or −). Red lines represent associations at p<0.01, and results with p<0.05 are also plotted in grey to show trends for additional phenotypes. “LN1” indicates the phenotype had 1 added to the variable, and then the variable was natural log transformed. The PheWAS phenotypes significantly associated with this SNP varied, from MI (known), to coronary artery disease and MI related phenotypes such as presence or absence of “percutaneous transluminal coronary angioplasty”, “angina”, and “coronary bypass surgery”.

Approximately one-fourth of the PAGE PheWAS results (26/111; 23%) represented SNP-phenotype associations in phenotype-classes closely related to previously known genotype-phenotype associations. For example, rs10757278 near *CDKN2A/CDKN2B* has been robustly associated with myocardial infarction (MI) [10], [11]. In this PheWAS, rs10757278 was associated with the “Cardiac” phenotype-class, but also with the related phenotype-classes of “Artery Treatment” and “Angina”. Specifically, rs10757278 was associated with phenotypes in the Artery Treatment phenotype-class, such as “percutaneous transluminal coronary angioplasty” (WHI, p = 2.86×10^−6^, β = −0.17, EA), and “coronary bypass surgery” (CHS, p = 9.60×10^−3^, β = −0.26, EA). The SNP rs10757278 was also associated with phenotypes in the Angina phenotype-class, such as presence or absence of angina (WHI, p = 6.59×10^−3^, β = −0.14, EA) and the phenotype “Ever see a doctor because of chest pain?” (ARIC, p = 4.44×10^−3^, β = −0.31, EA). Replication of association of this SNP with previously known phenotypes were also found with the phenotype-class “Cardiac”, with phenotypes such as “MI (Y/N)” (WHI, p = 1.39×10^−4^, β = −0.11, EA), and “MI status at baseline (Y/N)” (CHS, p = 6.35×10^−3^, β = −0.18, EA). Significant PheWAS associations at p<0.01 for rs10757278 are plotted by phenotype in Figure 2, as well as additional results at p<0.05.

**Figure 3:**
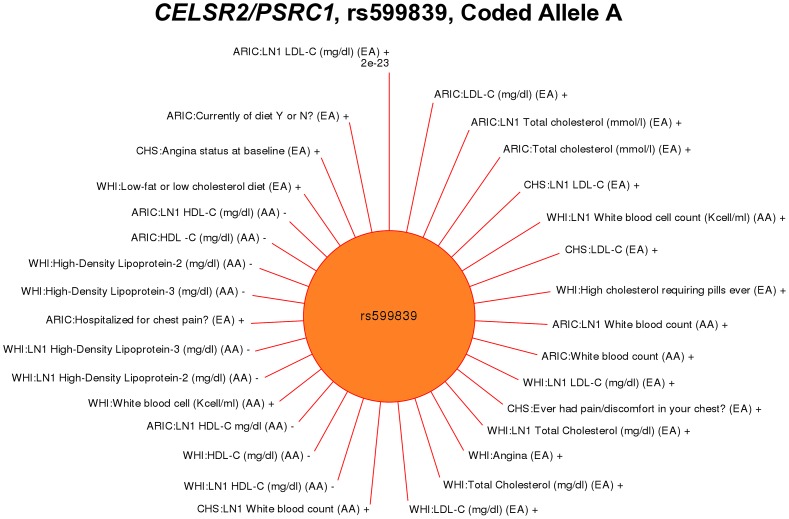
PheWAS associations for rs599839 near *CELSR2/PSRC1.* This SNP has previously published associations with serum LDL cholesterol levels, total cholesterol, and coronary artery disease. Genotype-phenotype associations are plotted clockwise starting at top for the association with the smallest p-value. The length of the line corresponds to –log10(p-value), the longer the line the more significant the result. The study race/ethnicity/and phenotype for each tests of association are listed. Red lines represent associations at p<0.01, and results with p<0.05 are also plotted in grey to show trends for additional phenotypes. “LN1” indicates the phenotype had 1 added to the variable, and then the variable was natural log transformed. The PheWAS phenotypes significantly associated with this SNP varied, from LDL cholesterol levels (previously published), to lipid level-related phenotypes such as “High cholesterol requiring pills ever”. In the case of coronary artery disease, phenotypes with significant results that were related to coronary artery disease included “Ever had pain/discomfort in your chest”, and “Hospitalized for chest pain”.

Another example of PheWAS associations for phenotype-classes closely related to known genotype-phenotype associations existed for rs599839 near the *CELSR2/PSRC1/SORT1* gene cluster. The SNP rs599839 has been associated with serum LDL cholesterol levels [8], [12]–[14], and coronary artery disease [13], [15]. In our PheWAS, associations were found for the “LDL-C” phenotype-class, as well the coronary artery disease related “Angina” and lipid related “HDL-C” phenotype-classes, including specific phenotypes such as “angina, presence or absence of” (WHI, p = 2.10×10^−4^, β = 0.25, EA), and “HDL-C” (WHI, p = 1.23×10^−3^, β = −0.04, AA). As expected, a significant association was also identified for the LDL-C level related phenotype “LDL-C (mg/dl)” (ARIC, p = 5.25×10^−22^, β = 6.42 EA). Significant PheWAS associations at p<0.01 for rs599839 are plotted by phenotype in Figure 3, as well as additional results at p<0.05.

### Potentially Novel Associations

PheWAS results were considered novel, if the significant phenotype-class associations varied substantially from the previously reported GWAS and candidate gene studies. Approximately one-third of the PAGE PheWAS results (33/111; 30%) represented novel genotype-phenotype-class associations. Further research will be required to determine the further validity of these exploratory results.

**Figure 4:**
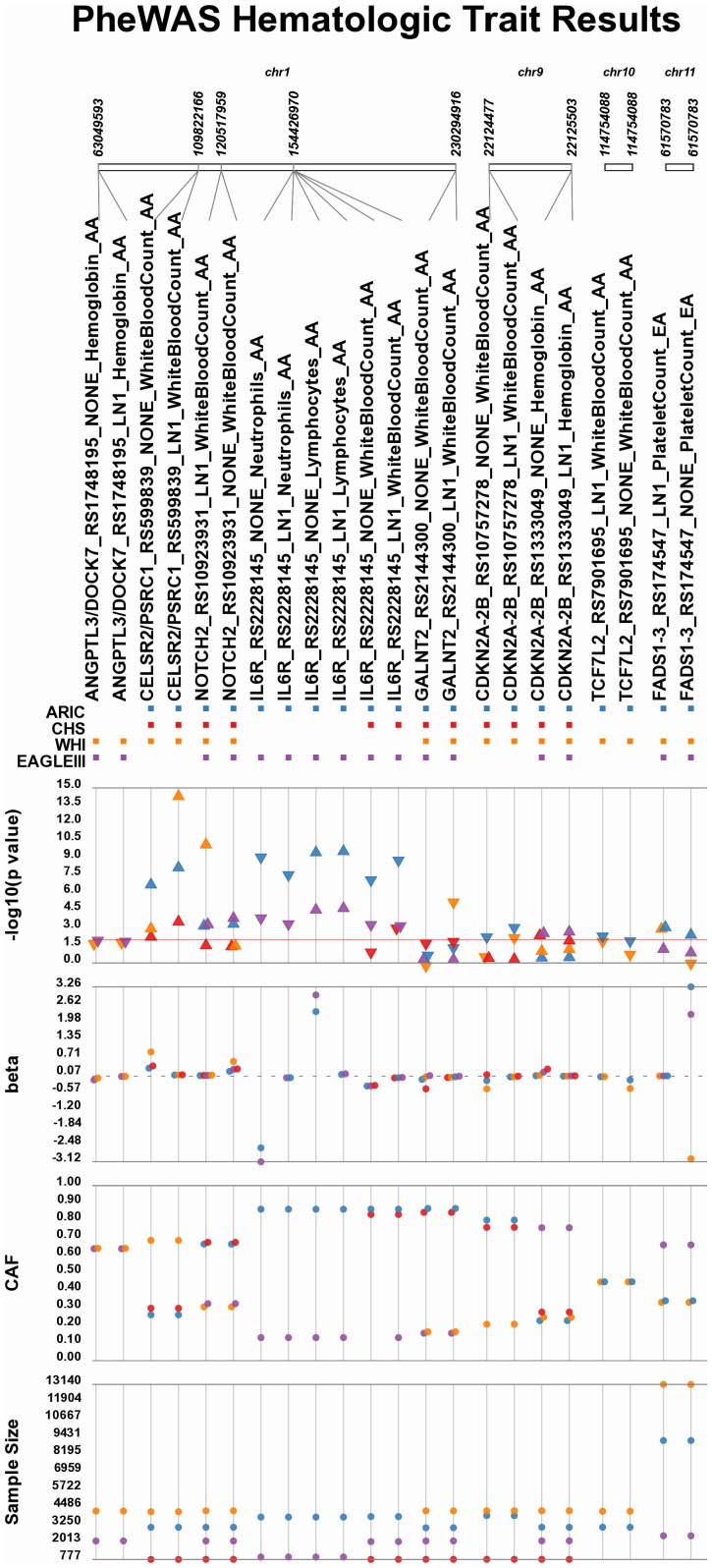
PheWAS results for blood cell counts and hemoglobin levels. Eleven novel genotype-phenotype-class associations were identified for white blood cell counts and hemoglobin levels collectively. The top track indicates the chromosomal location of each SNP, below that track is a SNP/Phenotype identification track containing the SNP ID, as well as the phenotype, phenotype transformation if present (LN1 = ln(1+variable)), and the race-ethnicity for the test population (AA or EA). The next track is a “presence/absence” track, box presence indicates if the SNP was present for ARIC (blue), CHS (red), WHI (orange), or EAGLE (purple). The next tracks are as follows: –log10(p-value), where the each p-value is plotted, the direction of the triangle indicates the direction of effect (triangle pointed up is positive, triangle pointed down is negative), base of the triangle corresponds to the location of the p-value, solid red line is positioned at p-value = 0.01; The next track is magnitude of effect (beta) dotted grey line is positioned at the null; Next are coded allele frequencies (CAF) for each study; Final track is sample size for each test of association.

The most statistically significant of the novel phenotype-class associations identified by this PheWAS include multiple associations involving phenotype-classes for hematologic traits in African Americans (Figure 4). SNPs rs599839 (*CELSR2/PSRC1*), rs10923931 (*NOTCH2*), rs2228145 (*IL6R*), rs2144300 (*GALNT2*), rs10757278 (*CDKN2A,CDKN2B*), and rs7901695 (*TCF7L2*) were each associated with white blood cell count phenotypes among AA (significant p-values ranging 7.96×10^−3^ to 9.99×10^−15^). *IL6R* rs2228145 was also associated with neutrophils and lymphocyte numbers in AA with p-values ranging from 2.44×10^−4^ to 4.66×10^−10^. These SNPs were previously associated with LDL-C, total cholesterol levels, and coronary artery disease (rs599839) [8], [12]–[14]; type 2 diabetes (rs10923931) [16]; C-reactive protein (rs2228145) [17]; coronary heart disease, HDL-C and triglycerides (rs2144300) [13]; MI (rs10757278) [11]; and type 2 diabetes (rs7901695) in EA [18]–[20]. It is likely that the majority of the significant findings for three of the SNPs on chromosome 1 [rs599839 (*CELSR2/PSRC1*), rs10923931 (*NOTCH2*), rs2228145 (*IL6R*)] are not truly novel given that these variants are likely in linkage disequilibrium with the white blood cell count-associated Duffy null allele (*DARC* rs2814778) [21], [22] in African Americans. Of note is *GALNT2* rs2144300 (p = 3.32×10^−6^ in WHI and 7.96×10^−3^ in CHS), located outside the 90 Mb region known to be associated with white blood cell counts in African Americans [21] and possibly representing a novel genotype-phenotype association for this trait. Also for chromosome 1, novel associations were identified in African Americans at p<0.01 for the phenotype-class “Hemoglobin” and *ANGPTL3* rs1748195, previously associated with triglycerides in European-descent populations [13], [19].

Of the remaining hematologic trait associations identified that were not on chromosome 1, rs10757278 near *CDKN2A/B* on chromosome 9 and *TCF7L2* rs7901695 on chromosome 10 were both associated with white blood cell count, neither of which were previously reported in GWAS for this trait [21], [22]. For *CDKN2A/B* rs1333049, a SNP previously associated with type 2 diabetes, coronary artery disease, and hypertension in European-descent populations [15], [23] p<0.01 associations were identified for the phenotype-class of Hemoglobin. Finally, a novel association in European Americans was noted between *FADS1* rs174547, a SNP previously associated with LDL-C [13], [19], and the phenotype-class of “Platelet Count” at p<0.01.

**Figure 5:**
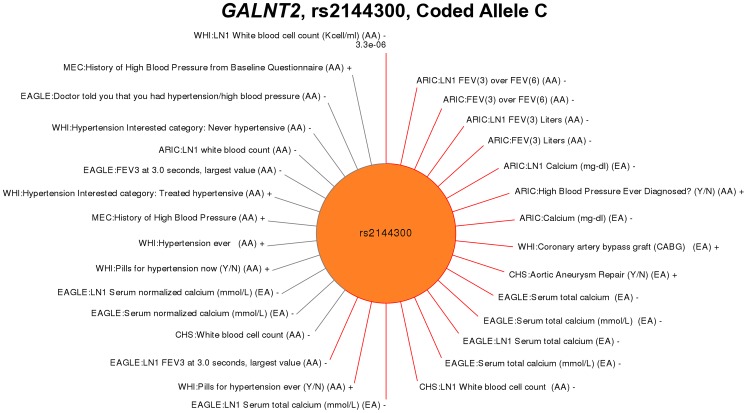
PheWAS associations for rs2144300 within *GALNT2*. The previously published associations for this SNP were with triglyceride and HDL cholesterol levels. Genotype-phenotype associations are plotted clockwise starting at top for the association with the smallest p-value. The length of the line corresponds to –log10(p-value), the longer the line the more significant the result. The study race/ethnicity/and phenotype for each tests of association are listed. Red lines represent associations at p<0.01, and results with p<0.05 are also plotted in grey to show trends for additional phenotypes. The novel PheWAS phenotypes significantly associated with this SNP varied, including white blood cell counts, forced vital capacity at three seconds (FEV3), and serum calcium levels.

Aside from hematologic traits, the most significant novel association identified in this PheWAS was identified for phenotypes in the phenotype-class “Forced Expiratory Volume in 3 Seconds (FEV3)” and *GALNT2* rs2144300 in African Americans (p-values ranging from 8.82×10^−3^ to 4.90×10^−4^). *GALNT2* rs2144300, previously associated with HDL-C in European Americans and African Americans [13], [24], has not previously been associated with lung function or asthma quantitative traits. Interestingly, *GALNT2* rs2144300 was also associated with phenotypes in the “Hypertension” phenotype-class among African Americans in this PheWAS Specifically the phenotypes were “High blood pressure ever diagnosed?” (ARIC, p = 1.61×10^−3^, β = 0.24) and “Pills for hypertension ever?” (WHI, 8.27×10^−3^, β = 0.15). Indeed, *GALNT2* rs2144300 displayed the most suggestion of pleiotropy among all the SNPs tested in this study. In addition to the associations identified in African Americans, rs2144300 was associated with phenotypes in the phenotype-classes “Serum Calcium” (p-values ranging from 1.47×10^−4^ to 8.10×10^−3^) and “Artery Treatment”, specifically the phenotypes “Coronary artery bypass graft (CABG)” (WHI, p = 2.46×10^−3^, β = 0.24) and “Aortic aneurysm repair” (CHS, 5.49×10^−3^, β = 0.57) in European Americans. Significant PheWAS associations at p<0.01 for rs2144300 are plotted by phenotype in Figure 5, as well as additional results at p<0.05.

The remaining significant novel PheWAS results have identified potentially pleiotropic effects for SNPs previously associated with lipid traits, type 2 diabetes, inflammation, myocardial infarction, and body mass index. The lipid trait-associated SNPs were associated with the “Menstruation” phenotype-class (specifically age at menarche) in European Americans (*CETP* rs3764261), the “Dieting” phenotype-class (*APOB* rs562338 in African Americans and *CELSR2/PSRC1/SORT1* rs599839 and rs646776 in European Americans), “Thyroid Goiter” in European Americans (*LIPG* rs2156552), “Artery Measurements” in European Americans (*LDLR* rs6511720) and “Artery Treatment” in African Americans (*PCSK9* rs11591147), “Plasma Serum Glucose Levels” (*APOE/APOC1/APOC4/APOC2/APOC3* rs4420638) in European Americans, and the “Angina” phenotype-class in European Americans (*CELSR2/PSRC1/SORT1* rs646776). For the type 2 diabetes-associated SNPs, the PheWAS-identified associations were observed for the phenotype-classes of “Dieting” (*IGFBP2* rs4402960) in European Americans, “Artery” and “Ever Smoked” (*ADAMTS9* rs4607103) in European Americans, “Hypertension” (*NOTCH2* rs10923931) in African Americans, “Heart Rate” (*LGR5* rs7961581) in European Americans, and “Menstruation” (specifically age at menarche) in European Americans (*FTO* rs8050136). Like type 2 diabetes-associated *ADAMTS9* rs4607103, BMI-associated *NEGR1* rs2815752 was associated with the phenotype-class of “Ever Smoked” in European Americans. The final two PheWAS-identified significant associations involved nutrient based phenotype-classes: MI-associated *CDKN2A/B* rs2383207 was associated with the phenotype-classes of “Vitamin B12” in European Americans, and inflammation-associated *IL6* rs1800795 was associated with the phenotype-class of “Carotene” in African Americans.

## Discussion

The PheWAS results herein present the result of tests of association between a large number of SNPs and an extensive range of phenotypes and traits available within five studies of the PAGE network. For this first PAGE PheWAS analysis we have emphasized associations that replicated across two or more independent PAGE studies for the same phenotype class and same race/ethnicity. Most of the robust findings reported here represent previously known genotype-phenotype relationships, but a tantalizing few also represent potentially novel pleiotropic relationships.

The 33 novel results presented here are intriguing, but it is important to emphasize that these first-pass analyses are considered hypothesis-generating, exploratory, and require additional scrutiny before the findings are further considered for follow-up, unlike the directed *a priori* hypothesis-testing analyses within PAGE that involve SNPs hypothesized to be associated with specific phenotypes. Further analysis of PheWAS results will be on an individual result basis and will include careful phenotype harmonization for traits and outcomes that cross two or more PAGE studies, as well as considerable investigation of the possible effect of covariates such as age, sex, and environmental exposure(s) on the association between genetic variation and phenotypic outcome.

One of the many challenges for the interpretation of PheWAS results is dissecting the genetic effect observed among correlated phenotypes. In some cases, the relationship is likely attributable to a common biological process with known genetic contribution (e.g., body mass index and waist circumference). In other cases, the networks that exist between intermediary and/or outcome related phenotypes add complexity to interpreting association results. For instance, genetic variation may impact the variation of a single phenotype, but variation in that phenotype could then result in changes in other downstream phenotypes indirectly. Examples of added complexity include obesity leading to impaired immune function [28], and metabolic syndrome where there is a spectrum of risk factors that are all associated with increased risk of cardiovascular disease and type 2 diabetes [25]. As a result, significant associations between a genetic variant and many phenotypes could represent a network or cascade of events. This is a potential interpretation of results found for SNP rs10923931 (*NOTCH2*) in AA, where type 2 diabetes was the previously reported association for this SNP and the novel result was found for hypertension, and type 2 diabetes and hypertension are often a co-occurrence. Further analysis of individual PheWAS results is necessary to conclusively establish the impact of the relationship between phenotypes on significant SNP-phenotype associations.

With the large number of phenotype-genotype associations calculated, there will be an increase in type 1 error due to multiple testing. A Bonferroni correction could be used within each individual study to choose a cutoff for significance that controls for multiple hypothesis testing. However, this would not take into account the correlations that exist between the phenotypes in these studies that impact the assumption of independence between tests as well as the correlations between the genotypes.

For our first PAGE PheWAS analysis, we chose to seek replication of results across studies and required the same direction of effect as one approach to reduce the false discovery rate. Significant results can still be found by chance across more than one study. Multiple challenges arise when attempting to get a metric of the type 1 error rate across multiple studies. First, as with individual studies, correlations between phenotypes and previous associations for the SNPs are still present. Also, there are varying type 1 error rates depending on the number of studies available for seeking replication. Quantification of how many results were found with a p-value cutoff, and without a p-value cutoff, depending on the number of studies where replication could be sought (2, 3, 4, or 5) provides some information about the number of significant results we found, in Table 2. Table 1 has the total number of results with and without p-value cutoff for individual studies. It is important to note that in cases where replication could be sought in more than two studies, there were cases where the result replicated in 3 or more studies, further increasing our confidence in the result.

A potential limitation of this study is the granularity of phenotypes within our phenotype classes. The phenotypes within some phenotype classes are the same or extremely similar, such as white blood cell count measurements across studies. However, the phenotype class “Artery Treatment” is broad in terms of the types of phenotypes included, such as presence/absence aortic aneurysm repair and presence/absence of angioplasty of the coronary arteries. For some classes, the replicated results encompass more variation in the phenotypes captured, compared to other results. As a result, significant associations between a genetic variant and all phenotypes in a network may be present. PheWAS is an exploratory and hypothesis generating exercise, thus the choice was made to have a broader match for some groups of phenotypes in order to allow for those phenotypes to be part of the exploration of the data. In addition, misclassification of phenotypes when matching is possible, and thus can limit identification of significant associations across studies. Other potential limitations include sample size/power, study heterogeneity, and the SNPs selected for study. As shown in Table 1, there is much variability across independent PAGE studies. While each PAGE study is sizeable, individual tests of association may be underpowered depending on the availability of the genetic variant, phenotype class, and race/ethnicity. Tests of association that failed to reach statistical significance may represent underpowered genotype-phenotype relationships and will require larger epidemiologic or clinic-based samples to identify. In regards to the potential impact of heterogeneity, we have some cases where replication existed in only two or three studies out of those where replication could be sought. In some instances this may be due to power, but this also may reflect the heterogeneity between studies, such as how various phenotypes are measured in individual studies and variation in mean age across the different studies. Finally, SNPs were originally selected for this study to replicate known genotype-phenotype associations and to generalize them to diverse populations. A comprehensive set of genome-wide“agnostic” SNPs may uncover additional pleiotropic or novel genotype-phentoype relationships not tested here.

Despite the the limitations present for this PheWAS, there are multiple strengths within our study. We have had the opportunity to perform a PheWAS of substantial size with an unprecedented diversity of high quality phenotypic measurements and traits, across multiple races/ethnicities. In addition, because of this PheWAS was conducted across multiple independent studies, we were able to identify the most robust genotype-phenotype relationships across studies

### Conclusion

This initial PheWAS within PAGE has presented challenges in terms of generating high-throughput tests of association across large epidemiologic studies as well as the synthesis of the resulting data and its eventual interpretation. Even with these limitations, this PheWAS demonstrates the utility of investigating the relationship between genetic variation and an extensive range of phenotypes by validating known genotype-phenotype associations as well as identifying novel genotype-phenotype associations, revealing complex phenotypic relationships and perhaps actual pleiotropy. The utility of this hypothesis-generating approach will continue to improve over time as more samples, variants, and phenotypes/traits across diverse populations are available for study in PAGE and other genomic resources. Larger, richer datasets coupled with methods development promise to more fully reveal the complex nature of genetic variation and its relationship with human diseases and traits.

## Methods

### Study Populations

All studies were approved by Institutional Review Boards at their respective sites (details are given in Text S1). The Population Architecture using Genomics and Epidemiology (PAGE) study includes the following epidemiologic collections: Atherosclerosis Risk in Communities (ARIC), Coronary Artery Risk in Young Adults (CARIDA), Cardiovascular Health Study (CHS), the Multiethnic Cohort (MEC), the National Health and Nutrition Examination Surveys (NHANES), Strong Heart Study (SHS), and Women's Health Initiative (WHI). For this PheWAS, data were available from ARIC, CHS, MEC, NHANES III, NHANES 1999–2002, and WHI (Table 1). The PAGE study design is described in Matise et al [26] and the PAGE PheWAS study design is described in Pendergrass et al [1].

### SNP Selection and Genotyping

All SNPs considered for genotyping in PAGE were candidate gene or GWAS-identified variants for phenotypes and traits available in the epidemiologic collections accessed by PAGE study sites. Cohorts and surveys were genotyped using either commercially available genotyping arrays (Affymetrix 6.0, Illumina 370CNV BeadChip), and/or custom mid- and low-throughput assays (TaqMan, Sequenom, Illumina GoldenGate or BeadXpress). Quality control was implemented at each PAGE study site independently. Study specific genotyping details are described in Text S1.

In this PheWAS, data were available for SNPs previously associated with HDL-C, LDL-C, and triglycerides [27], body mass index, obesity [28], type 2 diabetes, glucose, insulin [29], and measures of inflammation (C-reactive protein), among other diseases/traits. A total of 83 SNPs overlapped across at least PAGE study sites: ten were specifically selected for body mass index traits replication, three for C-reactive protein, six for coronary/cardiac traits, three for gout/kidney, 41 for lipids, and 20 for type 2 diabetes. Table S1 lists these SNPs, along with references reporting phenotypic associations from the NHGRI GWAS catalog [30] and the open access database of GWAS results of Johnson et al. 2009 [31]. The NHGRI GWAS catalog was most recently accessed in October, 2011. If no references were available from either of those two sources, a PubMed search was performed to retrieve relevant citations.

### Statistical Methods

All tests of association were performed independently by each PAGE study site using the following analysis protocol: Linear or logistic regressions were performed for continuous or categorical dependent variables, respectively, assuming an additive genetic model (0, 1, or 2 copies of the coded allele). For variables with multiple categories, binning was used to create new variables of the form “A versus not A” for each category, and logistic regression was used to model the new binary variable. Linear regressions were repeated following a y to log (y+1) transformation of the response variable with +1 added to all continuous measurements before transformation to prevent variables recorded as zero from being omitted from analysis. All analyses were stratified by race-ethnicity.

Test of association were calculated for the number of SNPs and phenotypes listed in Table 1. The software used to calculate the associations for each study was as follows: ARIC (StatSoftware), CHS (R [32]), MEC (SAS), MEC (SAS v9.2), WHI (R), EAGLE (SAS v9.2 using the Analytic Data Research by Email (ANDRE) portal of the CDC Research Data Center in Hyattsville, MD).

All association results from the tests of association were reported in standardized templates designed by the PAGE coordinating center to facilitate data sharing. All results were then imported into a relational database (MySQL). The database was also used to match previously reported GWAS data with the SNPs analyzed in this study.

### Plotting Significant Results

The software PheWAS-View was developed for data visualization of the PheWAS results as well as for plotting “Sun Plots” [33]. Synthesis-View [34], [35] was also used to present results within this manuscript. Both software packages are freely available software for academic users: http://ritchielab.psu.edu/ritchielab/software, and can be used with a web interface at: http://visualization.ritchielab.psu.edu/.

### Matching Phenotypes

**Figure 6:**
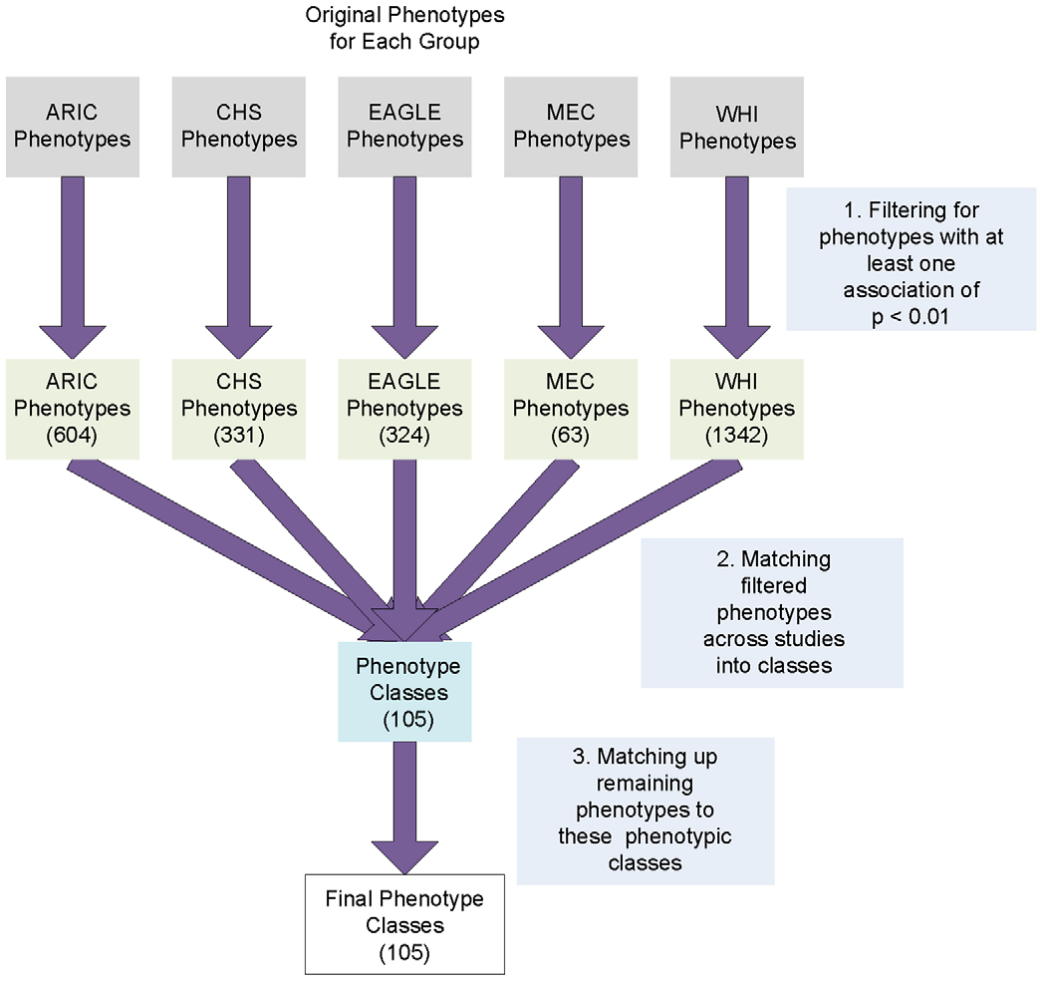
Workflow for phenotype matching, to develop the 105 phenotype classes. A MySQL database was used to filter the data from five studies for any results with p<0.01 to generate lists of the unique phenotypes for each individual PAGE study. The number of phenotypes that passed this significance threshold for each of the four groups was 604 (ARIC), 331 (CHS), 63 (MEC), 324 (EAGLE), 1,342 (WHI). Note that during the binning process, a smaller number of phenotypes are listed in Figure 6 than the total number of phenotypes referred to in the manuscript for the actual associations, in the phenotype matching process we only took into account distinct phenotypes regardless of whether or not they were transformed or untransformed or if they were categorical phenotypes binned into case/control phenotypes. Next, resulting phenotypes were then manually matched up between ARIC, CHS, MEC, EAGLE and WHI using and knowledge about the phenotypes and the known focus of specific PAGE study questions (such as arterial measurements including degree of arterial stenosis). In the last step, phenotypes from all studies, regardless of significance from genotype-phenotype tests of association, were matched to the already-defined phenotype classes using the criteria described above.

A total of 105 phenotype-classes were developed to manually match related phenotypes across studies. To bin related phenotypes into classes the following steps were used as visualized in Figure 6: First, using a MySQL database, the data from EAGLE, MEC, CHS, ARIC, and WHI were independently filtered for any tests of association results at p<0.01, and then lists of the unique phenotypes for each individual PAGE study were generated. The number of phenotypes that passed this significance threshold for each of the four groups was 604 (ARIC), 331 (CHS), 63 (MEC), 324 (EAGLE), 1,342 (WHI). Resulting phenotypes were then manually matched up between ARIC, CHS, MEC, EAGLE and WHI using knowledge about the phenotypes and the known focus of specific PAGE study survey questions (such as bone fracture questions used primarily for collecting information about osteoporosis). For some phenotypes, the specific phenotype existed clearly across more than one PAGE study, such as for the phenotype “Hemoglobin”, where hemoglobin measurements were present for ARIC, CHS, EAGLE, and WHI. Other groups of phenotypes that fell within similar phenotypic domains but were not represented in the same form across studies were also collected into phenotype classes. One example is the phenotypes grouped together for the phenotype class of “Allergy”. EAGLE collected specific quantitative data from allergy skin testing and had survey questions about the presence of allergies in participants. ARIC and MEC did not have skin allergy testing, but did have survey questions about the presence of allergies. Thus these allergy phenotypes were grouped together. Finally, phenotypes from all studies, regardless of significance from genotype-phenotype tests of association, were matched to the already-defined phenotype classes using the criteria described above. A phenotype that matched a phenotype class but was not associated with a SNP at the significance threshold of p<0.01 for a single study would still be included in the phenotype-class list. Using these criteria, a second curator reviewed the resultant phenotypes and phenotype classes for consistency and accuracy. To provide examples of the phenotype-classes, and which subphenotypes were matched with phenotype-classes, we show three phenotype-class examples in Table 5, and Table S2 contains the matched phenotypes across studies within the phenotype-classes for all phenotype-classes used within this study.

**Table 5:** Example phenotype-classes and binned subphenotypes within phenotype-classes.

Phenotype Class	Study	Sub-phenotype binned within the phenotype-class
Asthma	ARIC	Asthma ever diagnosed?
Asthma	ARIC	Chest wheeze, whistle alot?
Asthma	ARIC	Chest wheeze, whistle, otherwise?
Asthma	ARIC	Age at 1st wheezing attack
Asthma	ARIC	Age asthma started
Asthma	ARIC	Age asthma stopped
Asthma	ARIC	Short of breath wheezing attack?
Asthma	CHS	Inhaled steroids for asthma
Asthma	CHS	Asthma confirmed by doctor
Asthma	CHS	Current asthma diagnosis by doctor
Asthma	EAGLE	Doctor ever told you had: asthma
Asthma	MEC	Asthma: History of Asthma, Hayfever, Skin Allergy, Food Allergy or Any Other Allergy from Baseline Questionnaire
CRP	CHS	C-reactive protein, adjusted original values (mg/l)
CRP	CHS	C-reactive protein, original values (mg/l)
CRP	EAGLE	Serum C-reactive protein (mg/dL)
Hemoglobin	ARIC	Hemoglobin
Hemoglobin	CHS	hemoglobin (g/dl)
Hemoglobin	EAGLE	Hemoglobin (g/dL)
Hemoglobin	WHI	Hemoglobin (gm/dl)
Hemoglobin	EAGLE	Mean cell hemoglobin concentration
Hemoglobin	EAGLE	Mean cell hemoglobin: SI (pg)

Presented below are examples of phenotypes binned into three phenotype classes, “Asthma”, “BMI”, and “CRP”. Table S2 contains the complete list of matched phenotypes across studies within phenotype-classes, for all phenotype-classes used within this study.

It is important to note resources that can be used for further investigation of the phenotypes listed in Table S2, as well as in the results presented in this paper. The following study websites contain additional information about all collected study information, including how those phenotypes were collected:

- ARIC http://www.cscc.unc.edu/aric/
- CHS http://www.chs-nhlbi.org/CHSData.htm, https://biolincc.nhlbi.nih.gov/static/studies/chs/Other_Documents.htm
- WHI https://cleo.whi.org/data/Pages/home.aspx
- EAGLE http://www.cdc.gov/nchs/nhanes/nhanes_questionnaires.htm/
- MEC http://www.crch.org/multiethniccohort/mec_questionnaires.htm

### Criteria for Significance of Association

After creating phenotype-classes, significant PheWAS tests of association for single genotype-phenotype associations across PAGE studies were identified using a database query. Our criteria for considering a PheWAS test of association significant included a threshold of p<0.01 observed in two or more PAGE studies for the same SNP, phenotype class, and race/ethnicity and consistent direction of effect.

A total of 111 PheWAS tests of association met our criteria for significance (Table S3). Significant results were then binned based on class of association: known, related, and novel. In this PheWAS, *Known Associations* are positive controls and represent previously reported genotype-phenotype associations. *Related Associations* are SNPs significantly associated in this PheWAS with phenotypes judged to be closely related to phenotypes among Known Associations found here and the literature. *Novel Associations* are significant PheWAS results where 1) the association does not match a known association *and* 2) the phenotype for the PheWAS association is not within a similar phenotypic domain as the phenotype of known association.

### Ethics Statement

All participating studies were approved by their respective IRBs, and all study participants signed informed consent forms.

## Supporting Information

Table S1 The list of all SNPs available for two or more sites in this study, arranged by previously associated phenotypes.
(XLSX)

Table S2 A list of the study level phenotypes, the study from which the phenotype is available, and the phenotype-class for each phenotype that overlapped with another study.
(XLSX)

Table S3 The expanded results for the 111 PheWAS associations identified in this study.
(XLSX)

Table S4 The 52 PheWAS results that replicated previously published genotype-phenotype associations.
(XLSX)

Text S1 Information on study design, phenotype measurement, and genotyping for each study.
(DOCX)
