# Supplementary material for: Phenome-Wide Association Study (PheWAS) for Detection of Pleiotropy within the Population Architecture using Genomics and Epidemiology (PAGE) Network
Source: PLoS Genet. 2013 Jan 31;9(1):e1003087. doi: 10.1371/journal.pgen.1003087 (PMC3561060; doi:10.1371/journal.pgen.1003087)
Supplement: Text S1 — Information on study design, phenotype measurement, and genotyping for each study. (DOCX) [file pgen.1003087.s005.docx]

**Text S1**

| **Study** | **Genotyping Platform** |
| --- | --- |
| **ARIC** | *de novo* genotyping with TaqMan 6.0, or genome-wide association study data from the Affymetrix Genome-Wide Human SNP Array 6.0 |
| **CHS** | *de novo* genotyping with TaqMan 6.0, or genome-wide association study data from the  Illumina 370CNV BeadChip system |
| **EAGLE** | Illumina GoldenGate assay, Sequenom, Illumina Bead express, or genotypes were accessed using existing data in the Genetic NHANES database |
| **MEC** | OpenArray |
| **WHI** | Illumina’s Veracode GoldenGate genotyping assay |

**Descriptions of Participants, Data Collection, and Genotyping by Study:**

***Note: Ancestry/Race/Ethnicity was self-identified for all of the PAGE study sites.***

1. **Causal Variants Across the Life Course (CALiCo).**  CALiCo is a consortium of six demographically diverse population based studies and a central laboratory. This network contributes a maximum of approximately 58,000 men and women ranging in age from childhood to older adulthood. Two CALiCo studies are involved in this analysis: ARIC and CHS.
   1. **Atherosclerosis Risk in Communities (ARIC) Study.** The ARIC study is a multi-center prospective investigation of atherosclerotic disease in a predominantly bi-racial population [[1](#_ENREF_1)]. White and African American men and women aged 45-64 years at baseline were recruited from four communities: Forsyth County, North Carolina; Jackson, Mississippi; suburban areas of Minneapolis, Minnesota; and Washington County, Maryland. A total of 15,792 individuals participated in the baseline examination in 1987-1989, with follow-up examinations in approximate 3-year intervals, during 1990-1992, 1993-1995, and 1996-1998. After the institutional review board at every participating university approved the ARIC Study protocol, written informed consent was obtained from each participant.

***Genotyping:*** ARIC Study samples were genotyped using two approaches: *de novo* genotyping with TaqMan 6.0 (Applied Biosystems) and accessing previous genome-wide association study data from the Affymetrix Genome-Wide Human SNP Array 6.0 (Santa Clara, California). For the *de novo* genotyping data, the genotyping calls were made using the Applied Biosystem Autocaller 3.1 software. Internal QC's were included on every plate and across the full genotyping sample sets. QC genotypes were examined for consistency within the SNP genotyped. Other criteria include: 1) Internal genomic DNA, which is examined for replication across the study set; 2) Fingerprint blanks, which are used to identify the plate and verify cross-contamination and or sample error; 3) Genomic DNA pools to examine consistency of genotypes and plate validation; 4) No template controls (NTC), which serve as a background detector and review of any reagent problems; and 5) Autocaller confidence score, which determines percent genotyping call reliability. All SNPs are tested for departure from Hardy-Weinberg Equilibrium (HWE), and SNPs with HWE χ2>3.84 in unrelated cohorts (excluding duplicates) were excluded. SNP genotype data was also obtained from previous GWAS data. Genotyping was conducted using the Affymetrix Genome-Wide Human SNP Array 6.0 (Santa Clara, California). Sample exclusion criteria included discordant with previous genotype data, genotypic and phenotypic sex mismatch, suspected first-degree relative of an included individual based on genotype data (n=297), genetic outlier as assessed by Identity by State (IBS) using PLINK [[2](#_ENREF_2)], and >8 SD along any of the first 10 principal components in EIGENSTRAT [[3](#_ENREF_3)] with 5 iterations. Autosomal SNPs were used for imputation after exclusion of SNPs with HWE deviation p<5 x 10‾^5^, call rate <95%, or MAF<1%.

- 1. **The Cardiovascular Heart Study (CHS).** The CHS is a population-based longitudinal study of risk factors for cardiovascular disease in adults 65 years of age or older, recruited at four field centers (Forsyth County, North Carolina; Sacramento County, California; Washington County, Maryland; Pittsburgh, Pennsylvania) [[4](#_ENREF_4)]. Overall, 5,201 predominantly white individuals were recruited in 1989-1990 from random samples of Medicare eligibility lists, followed by an additional 687 African Americans recruited in 1992-1993 (total n=5,888).

***Genotyping:*** DNA was extracted from blood samples drawn on all participants at their baseline examination. Like ARIC, the CHS SNP genotypes were also obtained from two sources. First, *de novo* genotyping with TaqMan 6.0 (Applied Biosystems) accessing previous genome-wide association study data from the Affymetrix Genome-Wide Human SNP Array 6.0 (Santa Clara, California) was performed. The genotyping calls were made using the Applied Biosystem Autocaller 3.1 software. Internal QC's were included on every plate and across the full genotyping sample sets. QC genotypes were examined for consistency within the SNP genotyped. Other criteria included: 1) Internal genomic DNA, examined for replication across the study set; 2) Fingerprint blanks, which were used to identify the plate and verify cross-contamination and or sample error; 3) Genomic DNA pools to examine consistency of genotypes and plate validation; 4) No template controls (NTC), which served as a background detector and review of any reagent problems; and 5) Autocaller confidence score, which determined percent genotyping call reliability. All SNPs were tested for departure from Hardy-Weinberg Equilibrium (HWE), and SNPs with HWE χ2>3.84 in unrelated cohorts (excluding duplicates) were excluded. SNP genotype data was also obtained from previous GWAS data. The second source of genotyping data from CHS was performed at the General Clinical Research Center's Phenotyping/Genotyping Laboratory at Cedars-Sinai using the Illumina 370CNV BeadChip system. Genotypes were called using the Illumina BeadStudio software as above. The following exclusions were applied to identify a final set of 306,655 autosomal SNPs: call rate < 97%, HWE p < 1x10^-5^, > 1 duplicate error or Mendelian inconsistency (for reference CEPH trios), heterozygote frequency = 0, SNP not found in dbSNP.

1. **Epidemiologic Architecture for Genes Linked to Environment (EAGLE).** The EAGLE study accesses DNA samples and data collected for the National Health and Nutrition Examination Surveys (NHANES) by the National Center on Health Statistics (NCHS) at the Centers for Disease Control and Prevention (CDC). NHANES is a collection of diverse, population-based cross-sectional surveys of non-institutionalized Americans regardless of health status at the time of ascertainment. NHANES is considered a complex survey given that specific age groups (such as the elderly) and racial/ethnic groups (non-Hispanic blacks and Mexican-Americans) are oversampled. The NHANES data accessed for this work includes phase 2 of NHANES III (collected between 1991 and 1994). This survey contains DNA samples linked to demographic, health, and lifestyle data. Participants were consented by the CDC at the time of the survey and sample collection, and consent included the storage of data and biological specimens such as blood for future research [[5](#_ENREF_5)]. The present study was approved by the CDC Ethics Review Board. Because the study investigators did not have access to personal identifiers, this study was considered non-human subjects research by the Vanderbilt University Internal Review Board.

***Genotyping:*** In EAGLE, genotyping in NHANES III was performed using the Illumina GoldenGate assay (as part of a custom 384 OPA) by the Center for Inherited Disease Research (CIDR) through the National Heart Lung and Blood Institute’s Resequencing and Genotyping Service, or genotypes were accessed using existing data in the Genetic NHANES database, or genotyping was performed using Sequenom or Illumina BeadXpress. For the Sequenom/Illumina BeadXpress data, genotyping was performed in the Vanderbilt DNA Resources Core, where in addition to genotyping experimental NHANES samples, they genotyped blind duplicates provided by CDC and HapMap controls (n=360).

All EAGLE SNPs reported here passed CDC quality control metrics and are available for secondary analyses through NCHS/CDC. To ensure a minimal level of quality control, CDC requires investigators to genotype blinded duplicate samples specific to each dataset. For NHANES III, investigators are required to genotype four 96-well plates containing 16 blanks and 368 duplicate samples provided by CDC. For NHANES 1999-2002, investigators are required to genotype five 96-well plates containing 20 blanks and 402 duplicate samples provided by CDC. And, for NHANES 2007-2008, investigators are required to genotype three 96-well plates containing 12 blanks, 36 empty wells, and 240 duplicate samples provided by CDC. The threshold for concordance between blinded duplicate genotypes and the experimental NHANES DNA samples is 95%. Marker data below this threshold are not accepted by CDC and are returned to the investigator.

In addition to calculating blinded duplicate concordance, the CDC analyst also tests for deviations from Hardy-Weinberg Equilibrium (HWE) for each marker stratified by self-reported race/ethnicity. Marker data with two or more racial/ethnic groups out of HWE at p<0.0001 are not accepted by CDC and returned to the investigator.

1. **Multiethnic Cohort (MEC).** The MEC is a population-based prospective cohort study consisting of 215,251 men and women, and comprises mainly five self-reported racial/ethnic populations: African Americans, Japanese Americans, Latinos, Native Hawaiians and European Americans [[6](#_ENREF_6)]. The MEC was designed to provide prospective data on exposures and biomarkers potentially involved in cancer initiation and progression across groups with distinct cultural and dietary patterns. Between 1993 and 1996, adults between 45 and 75 years old were enrolled by completing a 26-page, self-administered questionnaire asking detailed information about dietary habits, demographic factors, level of education, personal behaviors, and history of prior medical conditions (e.g. diabetes). Between 1995 and 2004, blood specimens were collected from ~67,000 MEC participants at which time a short questionnaire was administered to update certain exposures, and collect current information about medication use. Study protocols and consent forms were approved by the institutional review boards at all participating institutions.

***Genotyping:*** Genotyping of 43 SNPs was conducted by the OpenArray platform (Life Technologies, Carlsbad, CA) at the Cancer Research Center in Hawaii following the recommended protocol. For all SNPs genotype call rates were >90% and individual call rates were >90%. Concordance rates between duplicate samples were 100%. HWE was examined and p-values were >0.01 in at least 4 of the 5 ethnic groups.

1. **Women’s Health Initiative (WHI).** WHI is a long-term national health study that focuses on strategies for preventing heart disease, breast and colorectal cancer and fracture in postmenopausal women. A total of 161, 838 women aged 50–79 yrs old were recruited from 40 clinical centers in the US between 1993 and 1998 [[7](#_ENREF_7)]. WHI consists of an observational study, two clinical trials of postmenopausal hormone therapy (estrogen alone or estrogen plus progestin), a calcium and vitamin D supplement trial, and a dietary modification trial. Trial exclusion criteria have been described previously [[8](#_ENREF_8)]. Study protocols and consent forms were approved by the institutional review boards at all participating institutions. A subset of 21,000 WHI women were selected for genotyping and inclusion in these PAGE analyses.

***Genotyping:*** In WHI, SNPs were genotyped at the Translational Genomics Research Institute (TGen) (Phoenix, AZ) on Illumina’s BeadXpress Reader using Illumina’s Veracode GoldenGate genotyping assay, following the manufacturer’s recommended protocol ([www.illumina.com](http://www.illumina.com)). Study protocol included calculation of concordance rates among duplicates, genotyping of HapMap samples, re-genotyping of failing samples, and other extensive QA procedures. All genotyping data reported here passed QA with individual and SNP call rates exceeding 95% and 97%, respectively.

1. (1989) The Atherosclerosis Risk in Communities (ARIC) Study: design and objectives. The ARIC investigators. American journal of epidemiology 129: 687-702.

2. Purcell S, Neale B, Todd-Brown K, Thomas L, Ferreira MA, et al. (2007) PLINK: a tool set for whole-genome association and population-based linkage analyses. American journal of human genetics 81: 559-575.

3. Price AL, Patterson NJ, Plenge RM, Weinblatt ME, Shadick NA, et al. (2006) Principal components analysis corrects for stratification in genome-wide association studies. Nature genetics 38: 904-909.

4. Fried LP, Borhani NO, Enright P, Furberg CD, Gardin JM, et al. (1991) The Cardiovascular Health Study: design and rationale. Ann Epidemiol 1: 263-276.

5. Prevention CfDCa (2002) National Health and Nutrition Examination Survey III (NHANES) DNA Specimens: Guidelines for Proposals to Use Samples and Proposed Cost Schedule. Federal Register 67: 51585-51589.

6. Kolonel LN, Henderson BE, Hankin JH, Nomura AM, Wilkens LR, et al. (2000) A multiethnic cohort in Hawaii and Los Angeles: baseline characteristics. American journal of epidemiology 151: 346-357.

7. Anderson GL, Manson J, Wallace R, Lund B, Hall D, et al. (2003) Implementation of the Women's Health Initiative study design. Ann Epidemiol 13: S5-17.

8. (1998) Design of the Women's Health Initiative clinical trial and observational study. The Women's Health Initiative Study Group. Control Clin Trials 19: 61-109.
